# Auxiliary Structures-Assisted Radiotherapy Improvement for Advanced Left Breast Cancer

**DOI:** 10.3389/fonc.2021.702171

**Published:** 2021-07-08

**Authors:** Runhong Lei, Xile Zhang, Jinna Li, Haitao Sun, Ruijie Yang

**Affiliations:** Department of Radiation Oncology, Peking University Third Hospital, Beijing, China

**Keywords:** breast cancer, auxiliary structures, plan optimization, dose distribution, IMRT, VMAT

## Abstract

**Background:**

To improve the quality of plan for the radiation treatment of advanced left breast cancer by introducing the auxiliary structures (ASs) which are used to spare the regions with no intact delineated structures adjacent to the target volume.

**Methods:**

CT data from 20 patients with left-sided advanced breast cancer were selected. An AS designated as A1 was created to spare the regions of the aorta, pulmonary artery, superior vena ava, and contralateral tissue of the upper chest and neck, and another, designated as A2, was created in the regions of the cardia and fundus of the stomach, left liver lobe, and splenic flexure of the colon. IMRT and VMAT plans were created for cases with and without the use of the AS dose constraints in plan optimization. Dosimetric parameters of the target and organs at risk (OARs) were compared between the separated groups.

**Results:**

With the use of AS dose constraints, both the IMRT and VMAT plans were clinically acceptable and deliverable, even showing a slight improvement in dose distribution of both the target and OARs compared with the AS-unused plans. The ASs significantly realized the dose sparing for the regions and brought a better conformity index (*p* < 0.05) and homogeneity index (*p* < 0.05) in VMAT plans. In addition, the volume receiving at least 20 Gy (V_20_) for the heart (*p* < 0.05), V_40_ for the left lung (*p* < 0.05), and V_40_ for the axillary-lateral thoracic vessel juncture region (*p* < 0.05) were all lower in VMAT plans.

**Conclusion:**

The use of the defined AS dose constraints in plan optimization was effective in sparing the indicated regions, improving the target dose distribution, and sparing OARs for advanced left breast cancer radiotherapy, especially those that utilize VMAT plans.

## Introduction

Female breast cancer has become the most common cancer worldwide in 2020, as estimated by the International Agency for Research on Cancer (IARC) (1). Adjuvant radiotherapy is essential for patients with advanced breast cancer who have undergone modified radical mastectomy. The standard target area for advanced breast cancer radiotherapy includes the chest wall and local lymph node regions (2). The percentage of breast cancer with lymph node involvement is approximately 27% (3), and the most common treatment principle is to irradiate adjacent lymph node regions along with the primary site after surgery. A study focusing on the anatomic pattern of nodal recurrence in breast cancer patients with radiotherapy indicated that the majority of nodal recurrence occurred in the axilla (42%), internal mammary nodes (32.5%), and supraclavicular nodes (25.5%) (4). This result indicates the need for a better dose distribution, especially in regional node areas.

For breast cancer patients after radiotherapy, mitigating the probability and severity of late toxicities is vital. This has been well documented by Brownlee (5), radiation to the chest wall and regional nodal areas can negatively impact the long-term cosmetic outcome of the irradiated area and cause severe complications due to incidental dosage to surrounding normal tissues, including the heart, lungs, and contralateral breast. Late cardiac toxicity and secondary malignancies are two major issues. It has been reported that the mean whole heart dose is linearly related to the myocardial infarction risk after radiotherapy, even if the mean whole heart dose is less than 2 Gy (6). A large population-based cohort study indicated that the long-term risk of ischemic heart disease after adjuvant radiotherapy in patients with left-sided breast cancer was higher than that in patients with right-sided breast cancer (7). The dose and volume of irradiated tissue are regarded as one of the key factors that strongly affect secondary cancer risk (8). Cancer risk after breast radiation therapy has been well estimated (9–11).

Advancing technologies for breast cancer radiotherapy, from standard three-dimensional conformal radiotherapy (3D-CRT) to intensity-modulated radiation therapy (IMRT) and volumetric modulated arc therapy (VMAT) have greatly mitigated the acute and long-term side effects by continually reducing the normal tissue dose. As reported, the mean heart dose was reduced from 5.4 Gy in the period of 2003-2013 (12) to 3.6 Gy in the period of 2014-2017 (13). For the ipsilateral lung, the average dose was 11.2 Gy in the period of 2010-2015 (14). For radiotherapy of patients with left-sided advanced breast cancer, 3D-CRT can cover the target with the expected dose, but fails to meet the dosimetric constraints for surrounding normal tissues (15–18). Compared to 3D-CRT, IMRT and VMAT with additional multiple beam directions, can produce better conformity and homogeneity on the target, especially in the lymph node regions, and can drastically reduce the volume of the heart and lung in the radiation field (19, 20). Although intermediate-high dose sparing for OARs was achieved, the low-dose radiation delivered to the lungs, heart, aorta, pulmonary trunk, and contralateral breast was compromised (12–14, 21).

Although the constantly updated IMRT and VMAT techniques provide preferable plans with better dose distribution for advanced left breast cancer radiotherapy, the use of some newer techniques developed during practice can greatly improve the outcome. For plan optimization, the realization of the desired dose distribution is based on an appropriate objective definition. These dose constraints rely on delineated structures, such as the PTV, left lung, right lung, and heart. However, for advanced left breast cancer radiotherapy, there is nearly no delineated structure that can be used to restrict dose in plan optimization in the regions of the aorta, pulmonary artery, superior vena cava, and contralateral tissues of the upper chest and neck. In addition, there is no delineated structure in the cardia and fundus regions of the stomach, left liver lobe, and splenic flexure of the colon; hence, an incidental dose to these areas can occur. Some practical and effective tips or approaches have been developed based on these planning techniques to shelter the aforementioned regions and achieve better OAR sparing. For example, rings around the target, with different thicknesses and locations, were used to restrict the dose distribution for the target and intermediate-high dose for the ipsilateral lung (22). Xu et al. used a blocking structure to block low doses in the lungs (23), while Lin et al. demonstrated the use of conformed rings as dose constraint structures for whole-breast radiotherapy planning (24). The two different rings can separately restrict the unwanted intermediate-high dose for the ipsilateral lungs and heart, and the low dose for the contralateral lung.

Hence, in the present study, we specifically created two different ASs, designated as A1 and A2, with different locations and dose constraints that were used in plan optimization to spare organs in these regions with no intact delineations and to further decrease the OAR dose and improve the target dose coverage as much as possible. Additionally, we used strict dose constraints to generate clinically acceptable and deliverable IMRT and VMAT plans. Finally, dosimetric differences between the two techniques were compared using the two ASs.

## Materials and Methods

### Patient Selection and Setup

A total of 20 patients with left-sided breast cancer were retrospectively selected for this study. Since this was a retrospective study, the need for informed consent was waived. These patients required the treatment of regional lymph nodes, including axillary and supraclavicular regional nodes (SRNs). Patients in this study had stage II–IV breast cancer and had undergone a mastectomy. Patients were simulated in the supine position with their arms over their heads and immobilized with an extended wing board with T-bar handgrip immobilization devices. CT images were acquired using a Philips Big Bore 16–slice scanner (Philips Medical Systems, Best, The Netherlands) with a slice thickness of 5 mm. The scanning range was stretched from the temporomandibular joint to the first vertebral body of the lumbar spine. During scanning, the patients had free respiration.

### Delineation of Structures

For each patient, the clinical target volume (CTV), which is consisted of the chest wall, axilla, and supraclavicular area, was delineated on planning CT scan by a specialized radiation oncologist, following the Radiation Therapy Oncology Group (RTOG) recommendations published in the RTOG breast contouring atlas. The PTV was formed by adding a 5 mm margin to the CTV in all directions, excluding the stomach and glenohumeral joint, while including the heart, colon, and skin in the chest wall region. A volume of less than 3 mm from the surface was cropped out. For OARs, the spinal cord, heart (outlined to the pulmonary trunk, including the pericardium and excluding major vessels), bilateral lungs, contralateral breast, liver, stomach, colon, esophageal inlet, trachea, and glenohumeral joint on the affected side were considered. The axillary-lateral thoracic vessel juncture region was also contoured for dose evaluation.

**Figure 1:**
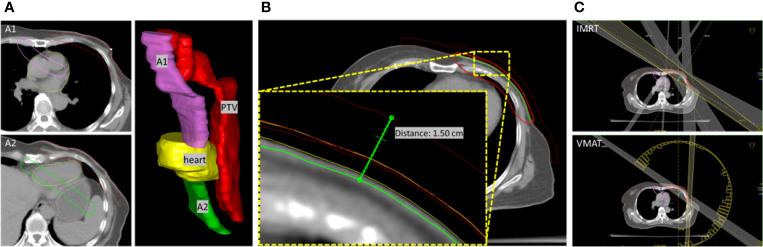
**(A)** The relative location of A1 and A2 to PTV and heart was presented in a 3D view. **(B)** Extended PTV and bolus for optimization. PTV-chest wall was extended 5 mm to the outside direction of the body, and a bolus with a thickness of 10 mm was defined. The red-line volume was the generated PTV (PTV-opt) used in optimization. Bolus was defined based on the outside of PTV-opt. Therefore, a total virtual bolus with a thickness of 1.5 cm was achieved in the optimization. **(C)** The beams setting for IMRT and VMAT plans.

Particularly, two ASs designated as A1 and A2 were contoured manually according to the distribution of the target and OARs, as shown in **Figure 1A**. Generally, these ASs were established to spare the regions with structures that have no intact delineation. A1 was contoured in the region of the aorta, pulmonary artery, and superior vena cava. The objective was to constrain the dose in these regions, with a distance of approximately 2 cm from the target boundary, as well as the dose to the contralateral tissue of the upper chest. In the contralateral tissue of the neck with no intact delineation of structures, A1 was contoured with a distance of approximately 1.5 cm from the target boundary. In the region of the heart and lungs, contouring was not performed since the delineated structures can be used to constrain the dose. A2 was contoured parallel to the target with a distance of approximately 1.5 cm sparing the cardia and fundus of the stomach, left liver lobe and splenic flexure of the colon, which are usually not delineated. A detailed example of the contouring performed is presented in the **Supplementary Material**. Two to three additional slices of contouring of ASs were essential to constrain the dose expansion.

### Dose Prescription and Planning Objectives

Conventional fractionation was performed with 50 Gy in 25 fractions given to the left chest wall after mastectomy, as well as the axillary and supraclavicular lymph node regions. The primary aim of planning was to deliver 100% of the prescribed dose to 95% volume of the PTV. The volume receiving > 110% of the prescribed dose was constrained to below 10%. The dose parameters for OARs were likely to be limited to the thresholds reported in **Table 1**.

**Table 1:** OARs dose constraints during plan optimization.

OARs	Dose constraints
A1	D_max_ < 20 Gy
A2	D_max_ < 30 Gy
Spinal cord	D_max_ < 20 Gy
Heart	V_5_ < 40%, V_20_ < 5%, D_mean_ < 5 Gy
Left lung	V_5_< 45%, V_20_ < 22%
Right lung	D_max_ < 5 Gy
Liver	D_mean_ < 3.8 Gy
Right breast	D_max_ < 5 Gy
Stomach	D_max_ < 40 Gy, D_30_ < 2 cc
Colon	D_max_ < 45 Gy, D_30_ < 2 cc
Esophageal inlet	D_max_ < 50 Gy
Trachea	D_max_ < 50 Gy
Left glenohumeral joint	V_20_ < 10%

### Treatment Plan Optimization

Both IMRT and VMAT planning were completed in Oncentra^®^ TPS (version 4.3.0.410, Elekta) with a collapsed cone convolution (CCC) dose calculation algorithm and a dose grid of 3.0 mm. 6MV photons were used in all the IMRT and VMAT plans. The chest wall motion induced by free respiration and skin dose compensation was carefully considered. The PTV-chest wall was extended 5 mm to the outside direction of the body, and the mass density of the extended volume in the air was assigned as 1. A bolus with a thickness of 10 mm was generated based on the extended PTV and linked to the beam fields for optimization, as shown in **Figure 1B**. Another bolus with the same thickness and coverage was directly located on the body surface and was used for the final dose calculation. To yield steep dose gradients near the tumor edge and, by extension, limiting the dose to the surrounding tissues, After optimization, the extended PTV with the assigned mass density was set to disabled, and the final dose was calculated.

In the IMRT planning, five beam fields were arranged (295°–315°, 330°, 0°, 30°, and 120°–135°). The angles (295°–315° and 120°–135°) of the tangential beams were adjusted to cover the chest wall PTV while excluding the right breast tissue. The beams with angles of 330°, 0°, and 30° cover the entire PTV volume, as judged from the beam’s eye view. In the VMAT planning, dual arcs starting from 180° with a length of 235°–245° in the counter-clockwise direction were set up, as shown in **Figure 1C**. The gantry spacing was 4°, and the maximum delivery time was 100 s. The bolus is linked to each beam. The maximum number of iterations was 90, with the inhomogeneity correction enabled during dose calculation. The objective parameters were adjusted throughout the optimization to best meet the OAR dose constraints without compromising the PTV coverage mentioned above.

### The Plan Assessment

The following parameters were recorded and compared between IMRT and VMAT plans. For PTV, the D_max_ (2%), D_min_(98%), D_mean_, V_105_, V_110_, Vtp (PTV volume within the prescribed isodose surface), Vt (PTV volume), D_5%_, and D_95%_ were collected. The conformity index (CI) was measured using Vtp^2^/(Vt·Vp) (defined below), and the dose homogeneity index (HI) was measured as D_5%_/D_95%_. CI and HI were calculated, and the closer the CI and HI values to 1, the better the conformal coverage.

$$\text{CI}=\text{Vt}{\text{p}}^{2}/(\text{Vt}\cdot \text{Vp})$$

(Vtp: PTV volume within the prescribed isodose surface; Vt: PTV volume; Vp: prescription volume in the body)

$$\text{HI}={\text{D}}_{5\%}/{\text{D}}_{95\%}$$

(D_5%_: = minimum dose to 5% of the PTV, D_95%_ = minimum dose to 95% of the PTV)

To evaluate the irradiated dose to OARs, the analysis included the mean dose (D_mean_) and V_x_ (OAR the volume receiving at least x Gy), depending on the organ.

The collected dosimetric parameters for the heart were D_mean_, V_5_, V_10_, and V_20_; for the left lung, D_mean_, V_5_, and V_20_; and for the right lung D_mean_, D_max_ (2%), and V_5_. A two-tailed t-test was used to compare the parameters. Statistical significance was accepted for *p*-values < 0.05, which were assigned as * or ^†^.

## Results

### Dose Distribution in the Regions of A1 and A2

As expected, the use of A1 (D_max_ < 20 Gy) and A2 (Dmax < 30 Gy) in IMRT and VMAT plan optimization successfully constricted the extension of intermediate-high doses in the adjacent region of the target. As shown in **Table 2**, in the IMRT plans, the D_max_ of the A1 was significantly lower than that of the AS dose constrained plans (38.08 ± 6.87 *vs*. 21.41 ± 1.44; *p* < 0.05). Meanwhile, the D_mean_ of A1 was also significantly lower than that of the AS-unused plans (9.41 ± 2.59 *vs*. 7.62 ± 2.10; *p* < 0.05). The D_max_ and D_mean_ of A2 decreased slightly when the AS dose constraint was used. In the VMAT plans, the D_max_ and D_mean_ of the A1 were significantly lower when the AS dose constraint was used (36.92 ± 5.55 *vs*. 20.98 ± 0.83; *p* < 0.05; and 9.70 ± 2.28 *vs*. 8.36 ± 1.45; *p* < 0.05). For A2, the D_max_ was lower when the ASs dose constraint was used (28.75 ± 7.65 *vs*. 24.61 ± 5.66; *p* < 0.05). A slight reduction in the D_mean_ of A2 was also observed, but with no statistical significance. Compared with the IMRT plans, the use of ASs in VMAT plans reduced the D_max_ of A1 and A2 and the D_mean_ of A2.

**Table 2:** ASs dose constraint effect.

Plans	ASs	D_max_ (mean ± SD)		D_mean_ (mean ± SD)	
		ASs-unused	ASs-used	ASs-unused	ASs-used
IMRT	A1	38.08 ± 6.87	21.41 ± 1.44*	9.41 ± 2.59	7.62 ± 2.10*
	A2	27.34 ± 7.13	26.10 ± 4.59	12.12 ± 2.60	12.01 ± 2.35
VMAT	A1	36.92 ± 5.55	20.98 ± 0.83*	9.70 ± 2.28	8.36 ± 1.45*
	A2	28.75 ± 7.65	24.61 ± 5.66*	9.73 ± 2.33	9.44 ± 1.54

*p < 0.05, when the parameters were compared between the AS unused and AS groups.

**Figure 2:**
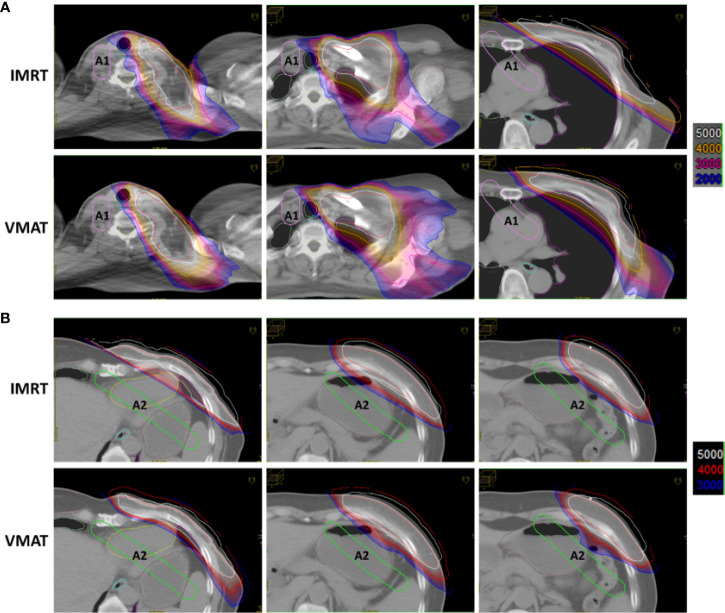
Auxiliary structures A1 and A2 reduced normal tissue dose in IMRT and VMAT plans. **(A)** Using A1, the organs in the indicated regions were protected from the intermediate-high dose. **(B)** Using A2, the organs in the indicated regions were protected from the intermediate-high dose.

For the two types of plans, the role of ASs in dose constriction was evaluated. As shown in **Figure 2A**, using A1, the organs, including the aorta, pulmonary artery, superior vena cava, and contralateral tissues of the neck, were protected from intermediate-high dose irradiation. The isodose line of 20 Gy (blue) was strictly constricted by the A1 structure. Using the auxiliary structure A2, as shown in **Figure 2B**, the cardia and fundus of the stomach, left liver lobe, and splenic flexure of the colon were protected from the intermediate-high dose. The isodose line at 30 Gy (blue) was strictly constricted by the A2 structure.

### Dosimetric Results of OARs

By introducing ASs in the regions with no intact contoured organs and using strict dose constraint parameters in plan optimization, the dose of OARs was relatively at a comparable level, even slightly lower than the AS-unused group as shown in **Table 3**, indicating the effective role of sparing the regions of A1 and A2 as well as the adjacent organs. For these two techniques, the mean whole heart dose was less than 5 Gy, and there was no significant difference for V_5_, V_10_, and V_20_, which were all at relatively low levels compared to previous studies (22, 25, 26). For the left-sided lung, the mean dose was slightly increased in VMAT plans compared with that in IMRT plans, from 11.31 Gy to 11.50 Gy and the V_20_ was also increased from 19.5% to 20.5%, both of which were also constrained to relatively low levels. However, there was no significant difference in V_30_ between the two techniques. In addition, V_40_ was lower in the VMAT plans than in the IMRT plans. For the right-sided lung and breast, both the mean dose and V_5_ were constrained to low levels. The V_40_ for the lymphedema-associated ALTJ region (41.04% *vs*. 27.22%, *p* < 0.05) was lower in the VMAT plans.

**Table 3:** Dosimetric results of OARs.

Organ	Parameter	IMRT (mean ± SD)		VMAT (mean ± SD)	
		ASs Unused	ASs Used	ASs Unused	ASs Used
Heart	D_mean_(Gy)	4.82 ± 0.47	4.70 ± 0.42	4.78 ± 0.15	4.74 ± 0.14
	V_5_(%)	16.41 ± 3.99	16.12 ± 4.17	19.46 ± 6.17	19.80 ± 5.00^†^
	V_10_(%)	8.21 ± 2.05	8.13 ± 1.78	8.71 ± 1.81	8.54 ± 1.88
	V_20_(%)	5.57 ± 2.04	5.36 ± 1.83	4.74 ± 1.39	4.54 ± 1.43*^†^
Lung L	D_mean_(Gy)	11.36 ± 0.58	11.31 ± 0.57	11.55 ± 0.37	11.50 ± 0.48
	V_5_(%)	45.84 ± 0.69	45.80 ± 0.69	46.32 ± 0.95	46.43 ± 1.37
	V_20_(%)	19.53 ± 1.68	19.53 ± 1.66	20.63 ± 0.93	20.49 ± 1.03
	V_30_(%)	13.94 ± 2.09	13.87 ± 2.00	13.87 ± 0.22	13.64 ± 1.04*
	V_40_(%)	9.42 ± 1.76	9.20 ± 1.72*	8.06 ± 0.94	7.86 ± 1.30^†^
Lung R	D_mean_(Gy)	1.32 ± 0.23	1.26 ± 0.24*	2.16 ± 0.17	2.10 ± 0.17^†^
	V_5_(%)	0.57 ± 1.29	0.21 ± 0.52	0.65 ± 0.61	0.54 ± 0.50^†^
Breast R	D_mean_(Gy)	0.90 ± 0.16	0.88 ± 0.15*	2.44 ± 0.22	2.32 ± 0.31^†^
	V_5_(%)	0.23 ± 0.49	0.14 ± 0.33	0.81 ± 0.73	0.64 ± 0.58^†^
ALTJ	D_mean_(Gy)	34.88 ± 9.93	35.22 ± 8.41	36.09 ± 4.12	35.58 ± 4.99
	V_40_(%)	46.32 ± 21.75	41.04 ± 22.51*	29.06 ± 17.32	27.22 ± 18.63^†^

*p < 0.05, when the parameters were compared between the AS unused and AS groups. ^†^ means p < 0.05, when the parameters were compared between IMRT and VMAT in the AS group.

### Dosimetric Results of PTV

**Figure 3:**
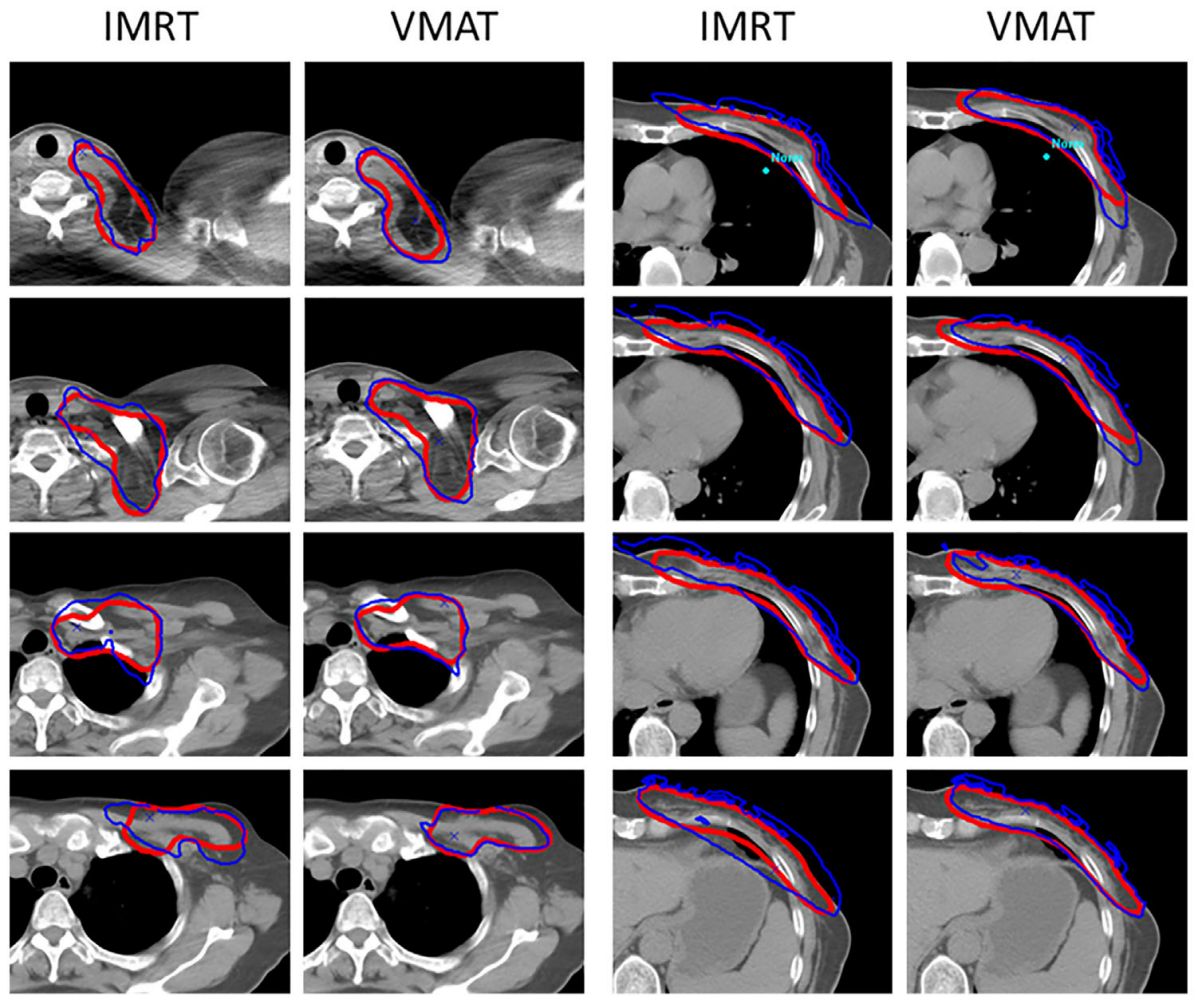
Dose distribution in the target with the use of ASs. VMAT plans presented more conformal dose distribution compared to the IMRT plans. The blue line means the 50 Gy isodose, and the red line means the outline of the PTV.

Except for OAR dose sparing, the use of A1 and A2 in plan optimization did not alter the plans’ clinical acceptance and delivery properties. As shown in **Table 4**, compared with AS-unused plans, the use of ASs significantly reduced the V_105_ for PTV (60.59 ± 10.43 *vs*. 53.72 ± 8.94, *p* < 0.05) in the IMRT plans. By comparison, the use of ASs in VMAT plans resulted in better target dose distribution. The mean maximum dose was reduced to 54.16 Gy from 55.49 Gy of IMRT plans. In addition, the mean minimum dose was constrained to 48.48 Gy instead of 47.46 Gy in IMRT plans. Under the condition that the prescription dose was 50 Gy, VMAT plans achieved a better mean dose of 52.30 Gy to the target. Furthermore, there has been a definite improvement in restricting high dose volume, as both the V_105_ and V_110_ were significantly reduced (53.72 ± 8.94 *vs*. 39.40 ± 12.47 and 5.03 ± 2.61 *vs*. 0.79 ± 1.24, *p* < 0.05). The conformity index (CI) was ameliorated from 0.70 to 0.79, and the homogeneity index (HI) was ameliorated from 1.12 to 1.08. As shown in **Figure 3**, the VMAT plan presented a more conformal target dose distribution in the supraclavicular and chest wall regions, as presented *via* the prescription isodose. In brief, with the use of ASs, the target dose distribution was improved and the VMAT plan execution was improved.

**Table 4:** Dosimetric results of target.

Parameter	IMRT (mean ± SD)		VMAT (mean ± SD)	
	ASs Unused	ASs Used	ASs Unused	ASs Used
D_max_ (2%, Gy)	55.64 ± 0.49	55.49 ± 0.43	54.06 ± 0.55	54.16 ± 0.68^†^
D_min_ (98%, Gy)	48.05 ± 0.70	47.46 ± 0.81*	48.82 ± 0.75	48.48 ± 0.91^†^
D_mean_ (Gy)	53.00 ± 0.43	52.70 ± 0.32*	52.31 ± 0.48	52.30 ± 0.35^†^
V_105_(%)	60.59 ± 10.43	53.72 ± 8.94*	36.81 ± 16.20	39.40 ± 12.47^†^
V_110_(%)	7.18 ± 5.66	5.03 ± 2.61	0.83 ± 2.99	0.79 ± 1.24^†^
CI	0.69 ± 0.04	0.70 ± 0.03	0.78 ± 0.03	0.79 ± 0.03^†^
HI	1.11 ± 0.01	1.12 ± 0.01*	1.07 ± 0.01	1.08 ± 0.02*^†^

*p < 0.05, when the parameters were compared between the AS unused and AS groups. ^†^p < 0.05, when the parameters were compared between IMRT and VMAT in the AS group.

### Delivery Efficiency

The total monitor unit (MU) of each plan partially reflects its delivery efficiency. As shown in **Table 5**, the average MU of each fraction for the IMRT and VMAT plans using ASs were 849.2 and 721.0, respectively. The VMAT technique significantly reduced the number of MU, indicating higher delivery efficiency.

**Table 5:** MU of IMRT and VMAT plans.

IMRT (mean ± SD)	VMAT (mean ± SD)
849.2 ± 80.1	721.0 ± 66.8*

*p < 0.05.

## Discussion

Continuous improvement of the radiotherapy plan quality to achieve better target dose distribution and the sparing of nearby critical structures can contribute to the superior radiotherapeutic response. Based on clinical practice during planning, various innovations were found and used, including the use of ASs. In this study, for advanced left breast cancer patient radiotherapy with modified radical mastectomy, we used two ASs, designated as A1 and A2, to spare the regions with no intact delineated structures. The dosimetric characteristics of the target and OARs were evaluated and compared based on this application in the IMRT and VMAT plans. The results indicated that the setting of A1 and A2 could protect the regions from intermediate-high dose irradiation substantially, and better performance was found in VMAT plans in the target dose distribution and OAR dose sparing.

The setting and use of the A1 auxiliary structure effectively constricted the expansion of the 20 Gy isodose line into the contralateral region of the neck, with a small probability of undergoing another radiotherapy in case of cancer in the right breast. In the region of the aorta, pulmonary artery, and superior vena cava, the A1 auxiliary structure effectively sheltered these organs from intermediate-high dose irradiation. The A2 auxiliary structure, located from the bottom of the heart towards the foot direction to the target edge, sheltered the cardia and fundus of the stomach, left liver lobe, and splenic flexure from intermediate-high dose irradiation. In particular, these organs are usually much closer to the target or even in an overlap, while the parts that are far away from the target are commonly not delineated. Hence, these separately distributed organs could not contribute to the formation of steep dose gradients near the tumor edge if they were used in plan optimization. In addition, the delineation of these organs would require more time and attention, but with low efficacy. Consequently, the setting and use of A1 and A2 ASs work very well in terms of convenience, practicability, and validity. Furthermore, if these ASs are automatically created before or after the target delineation, it will be better.

In the present study, we applied the ASs to the IMRT and VMAT plans to spare the intermediate-high dose for the defined regions, and the dosimetric results of the target and OARs were evaluated. The dosimetric results validated the availability and feasibility of the structures. Nonetheless, in the VMAT plans, the dose distribution parameters were better, which supports the combined use of the ASs and VMAT techniques. The improvement of target dose distribution, both in the local regional node area and primary chest wall, can potentially benefit advanced left breast cancer patients. The optimal trade-off between the target dose distribution and the surrounding OAR irradiation should be carefully analyzed and balanced when introducing new factors that can affect the outcome.

The high-level dose received on the axillary-lateral thoracic vessel juncture region causes lymphedema, which is a major quality of life concern (27–30). Enlarged dose volume should be avoided to reduce the risk of lymphedema. Therefore, improvement of target dose distribution, especially in the lymph node regions, can enable breast cancer patients to benefit more from radiotherapy. Among the radiotherapy techniques, VMAT significantly improved dose distribution in both conformity and homogeneity. In order to achieve a relatively low dose level for OARs while maintaining a better target dose distribution, the setting and use of the auxiliary structure was effective. In addition, a relatively stricter constraint on the parameters worked well. These constraints did not significantly deteriorate the quality of the target dose distribution when combined with the use of ASs in optimization. Apparently, a lower dose of OARs can bring more benefit to patients.

For patients who had undergone a mastectomy, the skin on the chest wall area was included in the planning target volume. Respiratory movement-induced tissue deformation-related dosimetric effects on both the target and OARs must be carefully considered (31–34). At present, various external treatment planning systems provide skin-flash tools or an optimization bolus for planning. PTV extending outside the skin contour and bolus located on the surface of the extended PTV are commonly used processing methods (35). When using IMRT and VMAT techniques, the perpendicular beam entrance direction may decrease the skin dose when compared to tangential beams owing to the decreased amount of scattered dose at the surface (36, 37). In this study, a bolus with a thickness of 10 mm was used. During optimization, the bolus was located on the extended PTV, and the another one was placed on the actual body surface for the final dose calculation. Hence, the opened beam field was maintained, and the actual dose was obtained.

It is quite clear that the quality of contouring accuracy and dosimetric compliance plays a critical role in modern radiotherapy. For planning techniques, improvement of target dose distribution and reduction of OAR dose suffering was the primary objective, and advanced planning strategies for breast cancer radiotherapy have greatly reduced the OAR dose suffering. For structure contouring, the anatomic pattern of nodal recurrence in breast cancer patients indicated the clinical demand for prescribed dose coverage in the presented regions (4).

## Conclusions

The setting and use of ASs, designated as A1 and A2, in plan optimization were effective in sparing the indicated regions, improving the target dose distribution, and reducing the normal tissue dose for advanced left breast cancer radiotherapy. The use of ASs in VMAT is recommended.

## Data Availability Statement

The original contributions presented in the study are included in the article/**Supplementary Material**. Further inquiries can be directed to the corresponding author.

## Author Contributions

RL and RY contributed conception and design of the study. RL, XZ, and HS organized the database. RL performed the statistical analysis and wrote the first draft of the manuscript. RL and JL wrote sections of the manuscript. JL and RY read and revised the manuscript. All authors contributed to the article and approved the submitted version.

## Funding

This study was partly supported by the Beijing Municipal Commission of Science and Technology Collaborative Innovation Project (Z201100005620012), Capital’s Funds for Health Improvement and Research (2020-2Z-40919), the Natural Science Foundation of Beijing (7202223), and the National Key Research and Development Program of China (2021YFE0202500).

## Conflict of Interest

The authors declare that the research was conducted in the absence of any commercial or financial relationships that could be construed as a potential conflict of interest.
