# Supplementary material for: Auxiliary Structures-Assisted Radiotherapy Improvement for Advanced Left Breast Cancer
Source: Front Oncol. 2021 Jul 8;11:702171. doi: 10.3389/fonc.2021.702171 (PMC8340769; doi:10.3389/fonc.2021.702171)
Supplement: Supplementary file 1 [file DataSheet_1.pdf]

A1 was contoured to spare the regions of the aorta, pulmonary artery, superior vena cava, and contralateral tissue of the upper chest and neck.

A2 was contoured to spare the regions of the cardia and fundus of the stomach, left liver lobe, and splenic flexure of the colon.

The thickness of A1 and A2 was 1 cm.

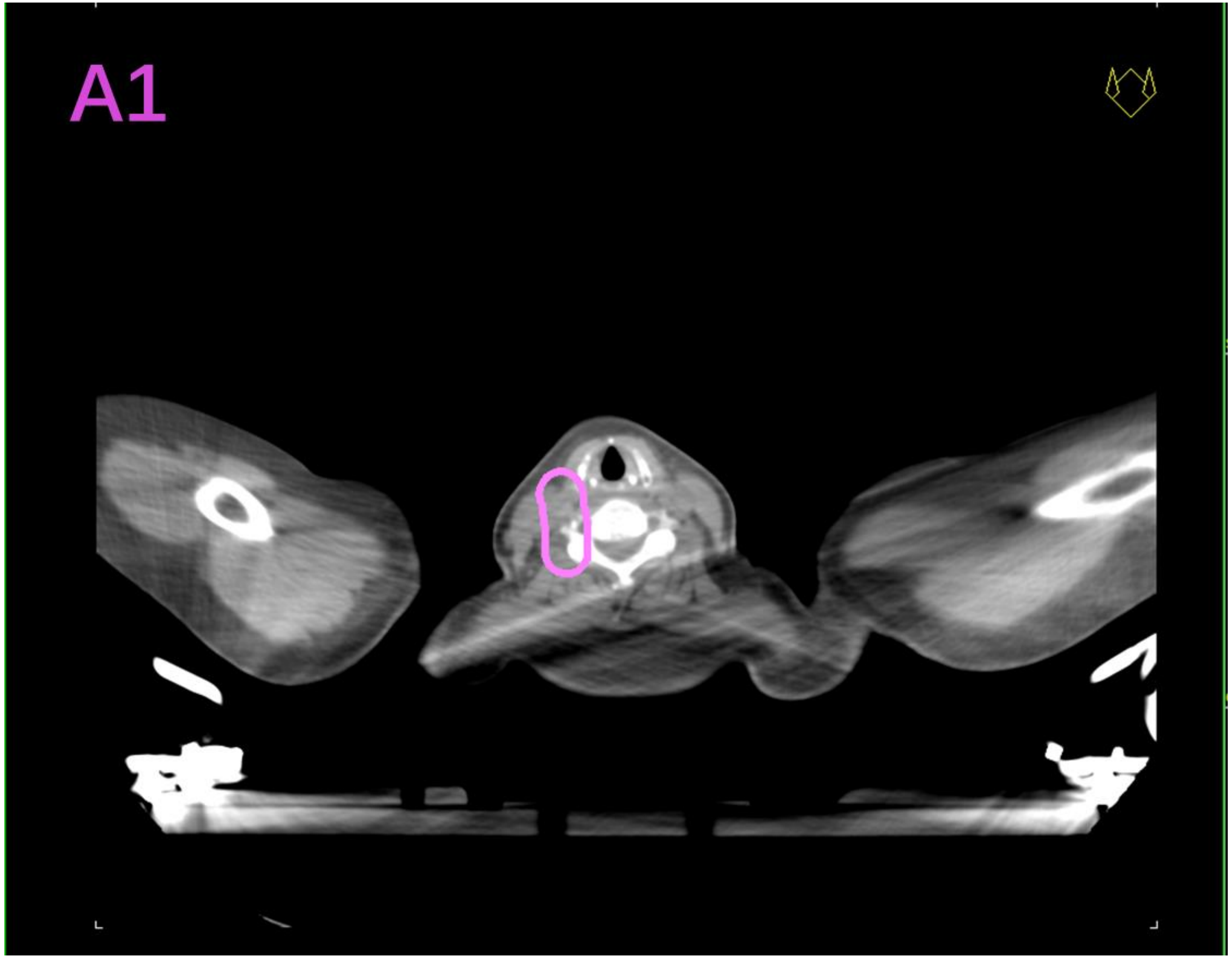
A1

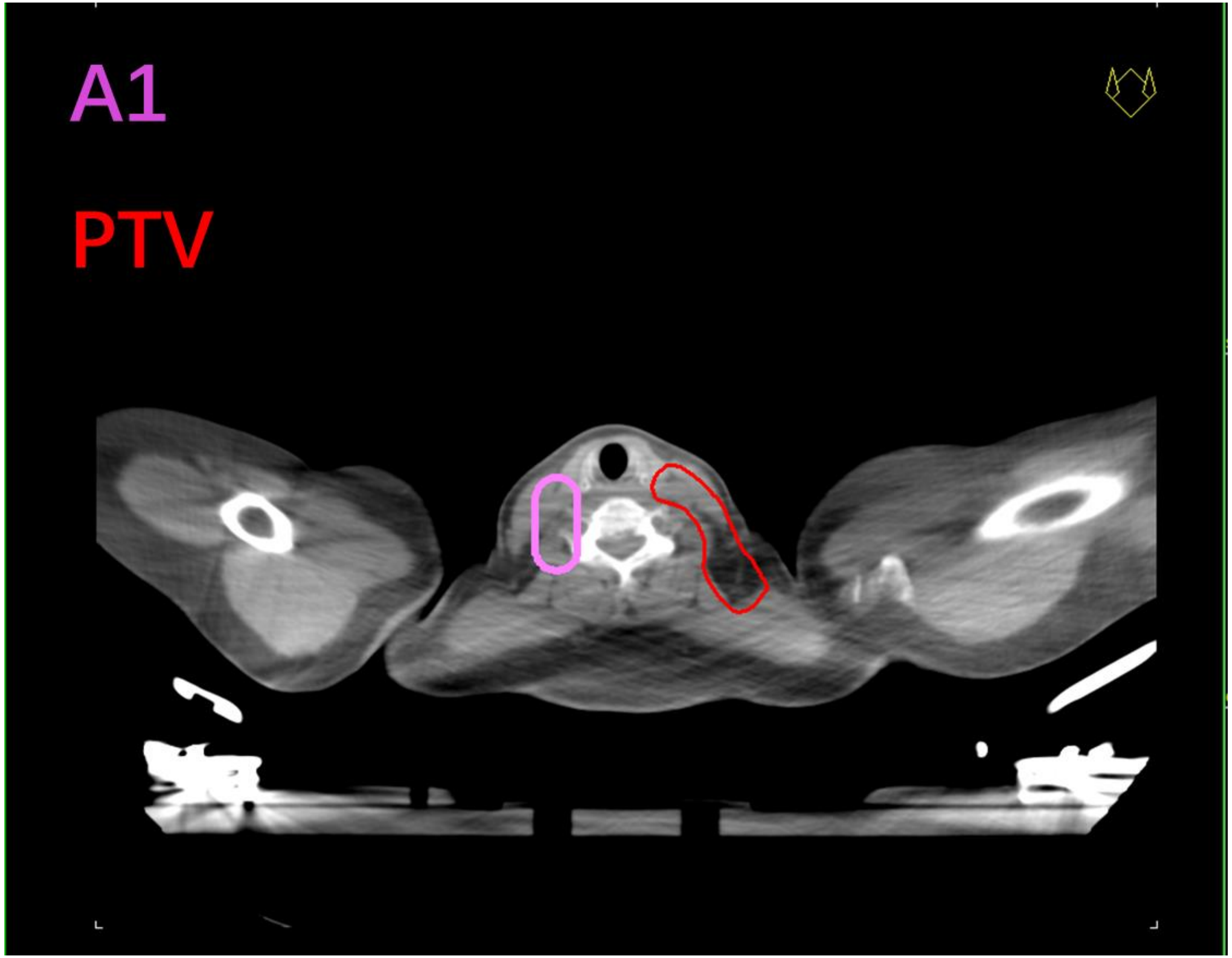
A1
PTV

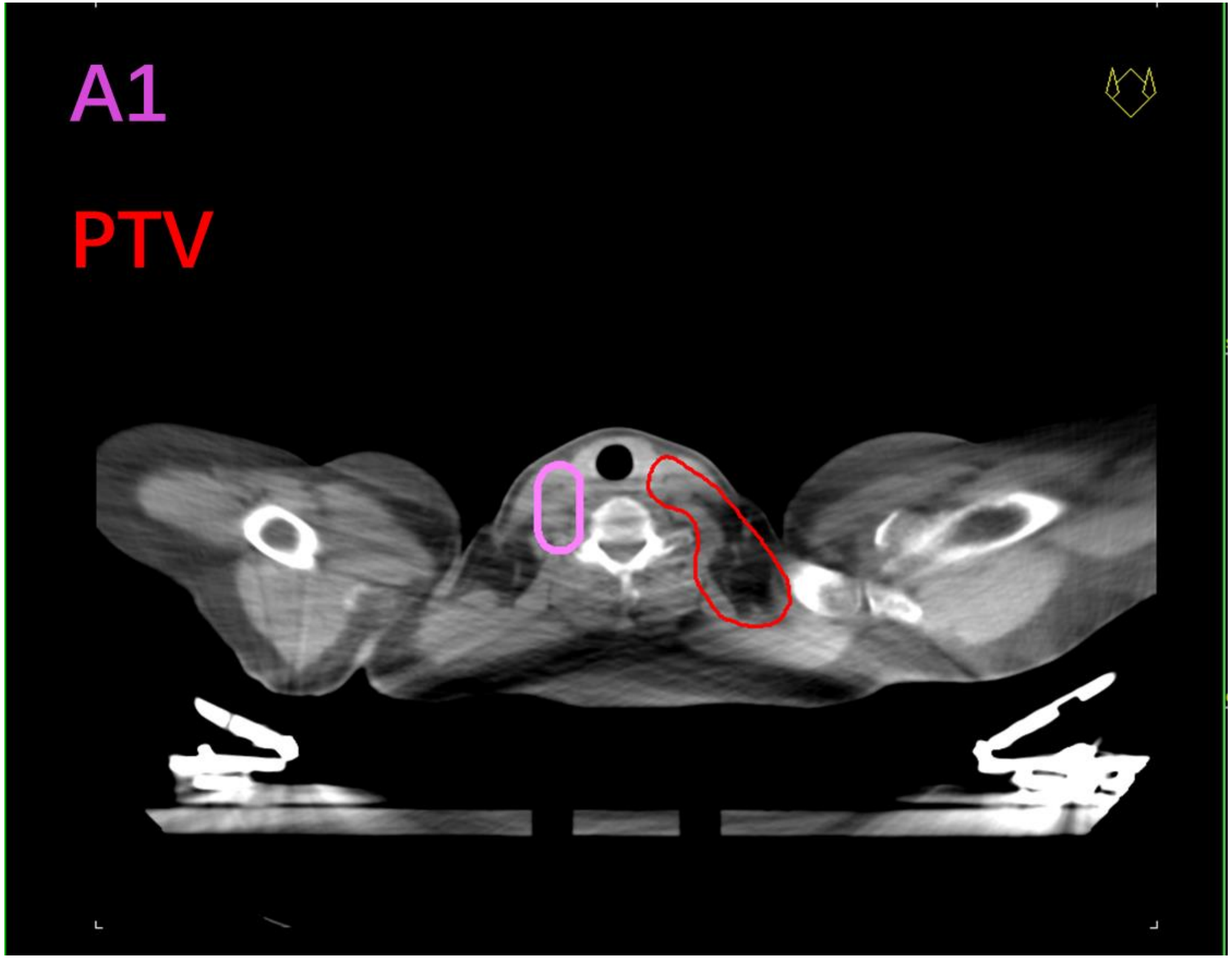
A1
PTV

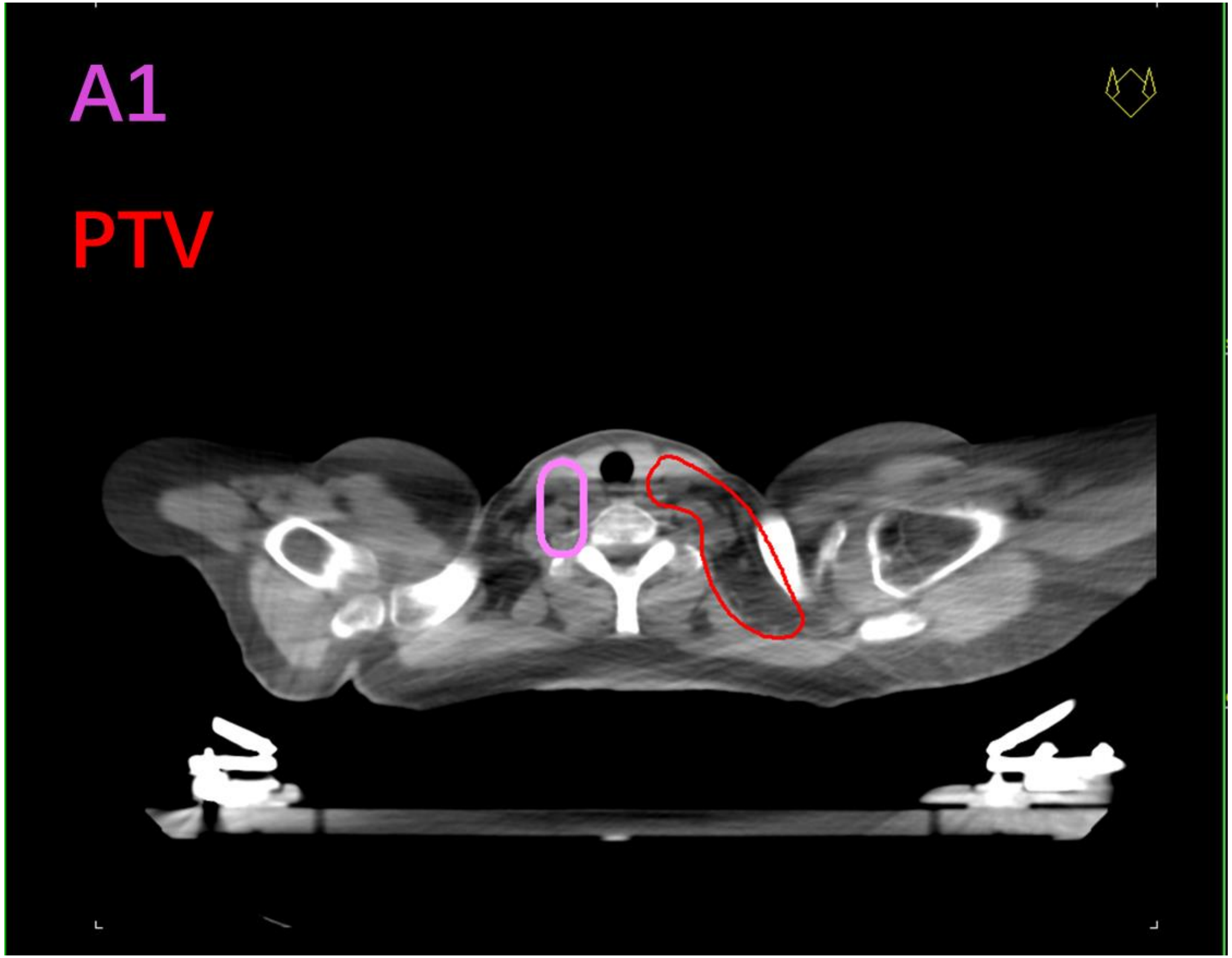
A1
PTV

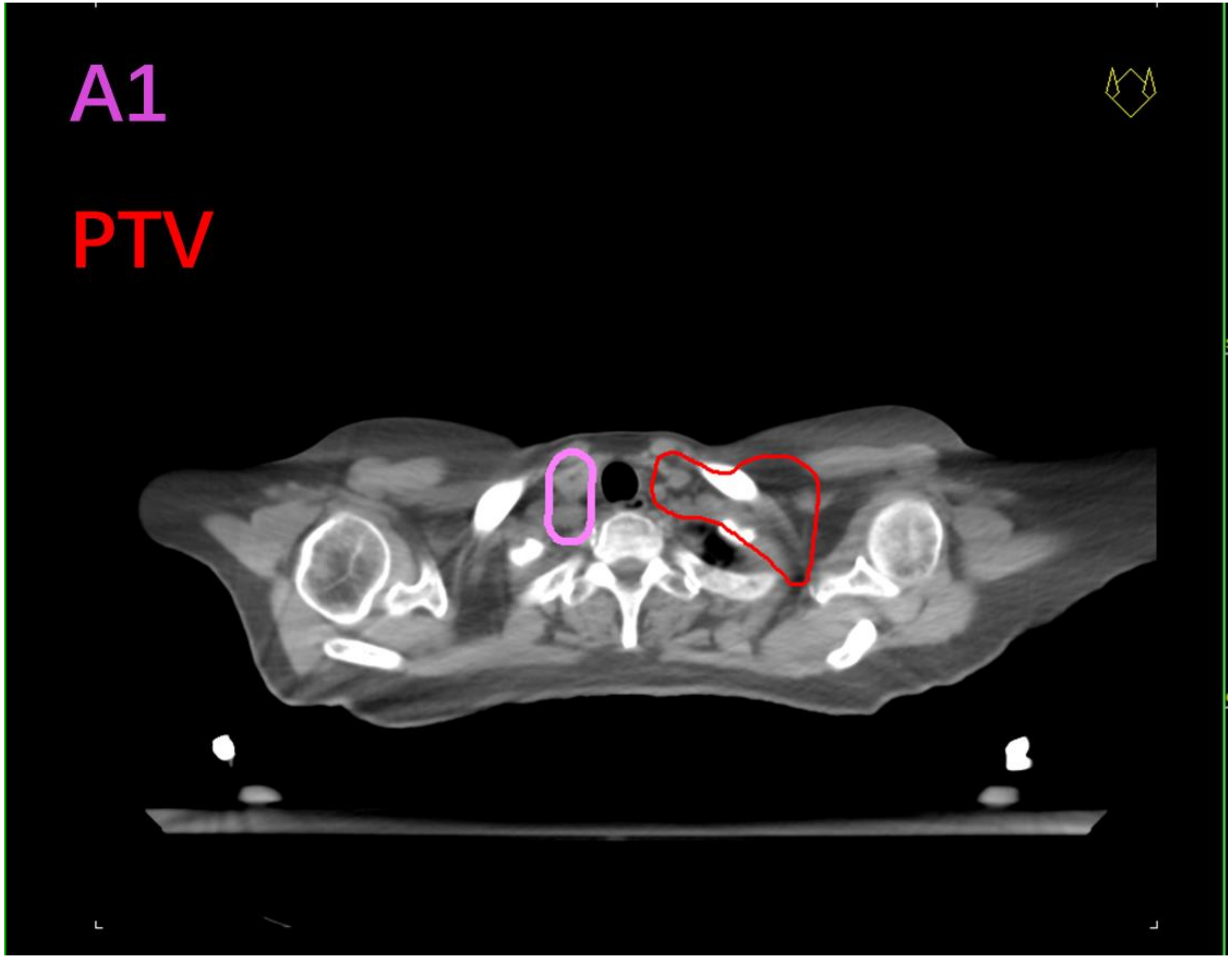
A1
PTV

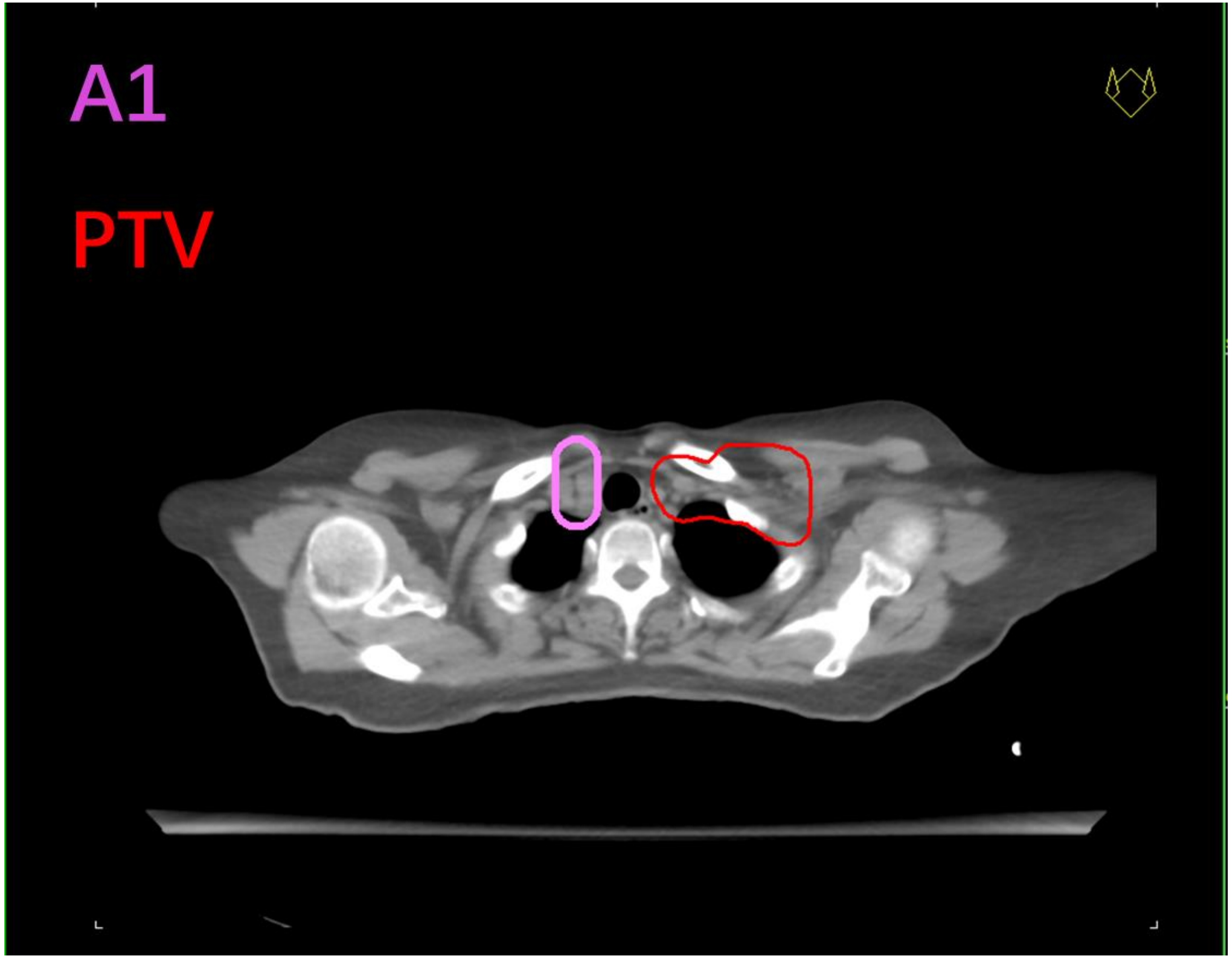
A1
PTV

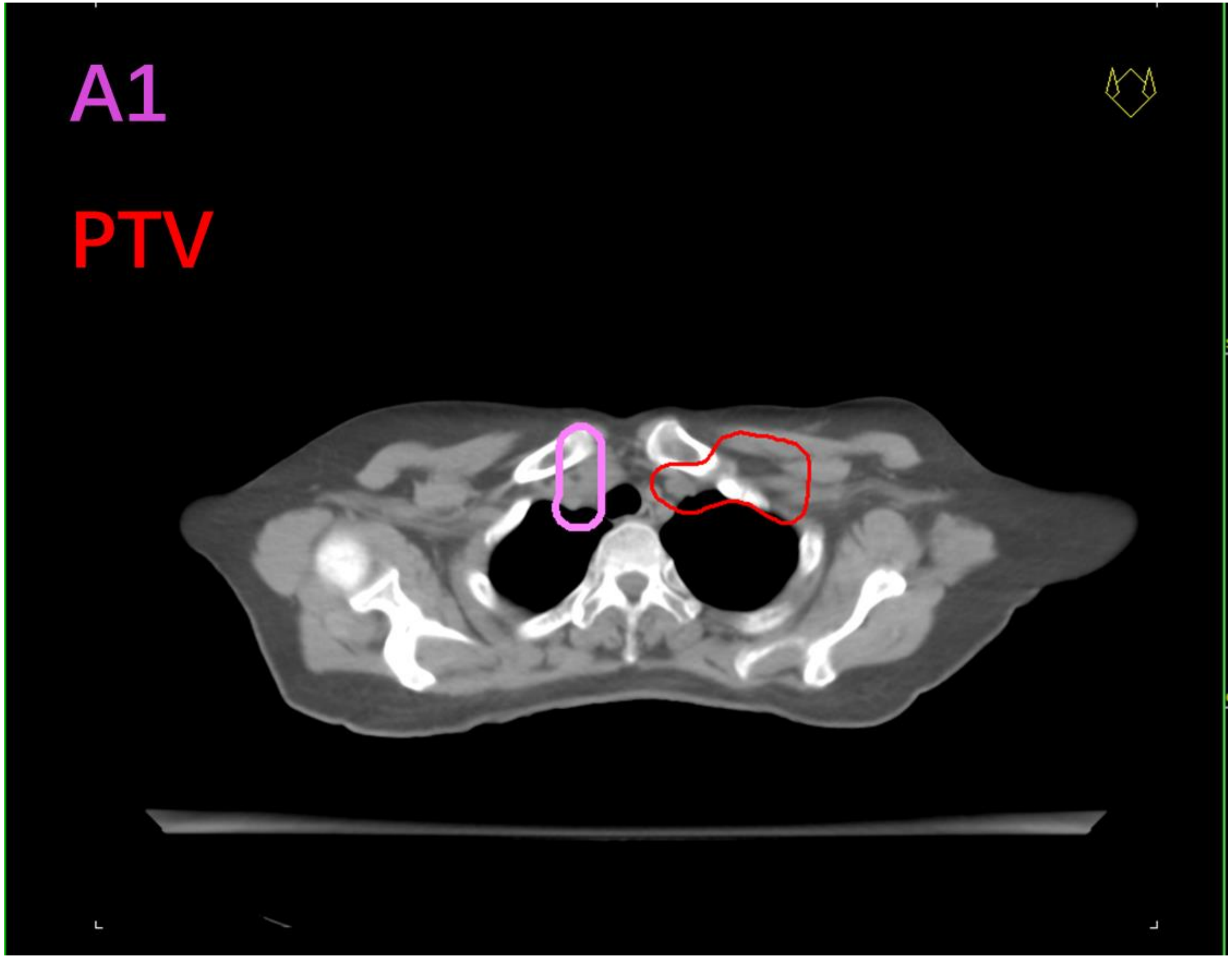
A1
PTV

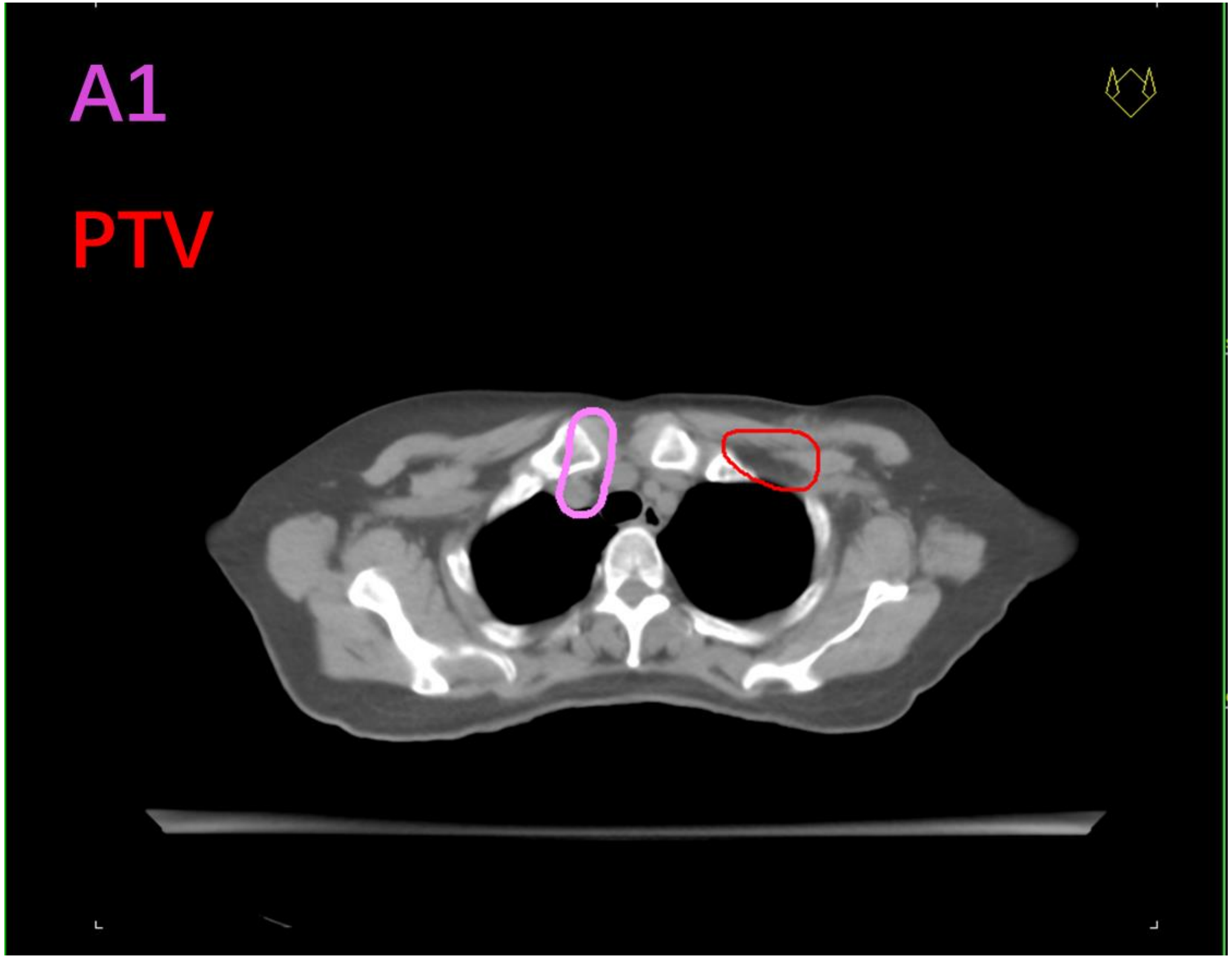
A1
PTV

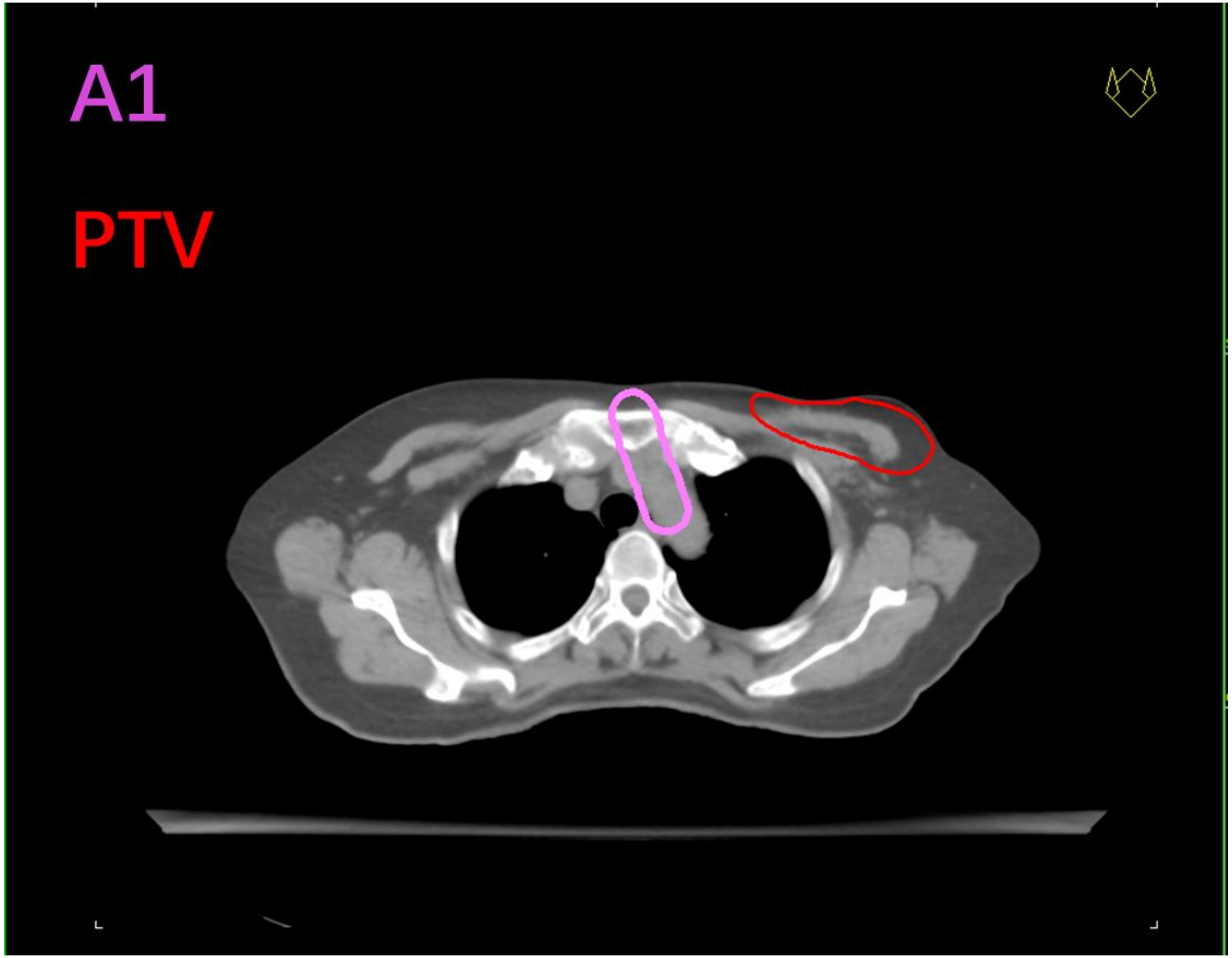
A1
PTV

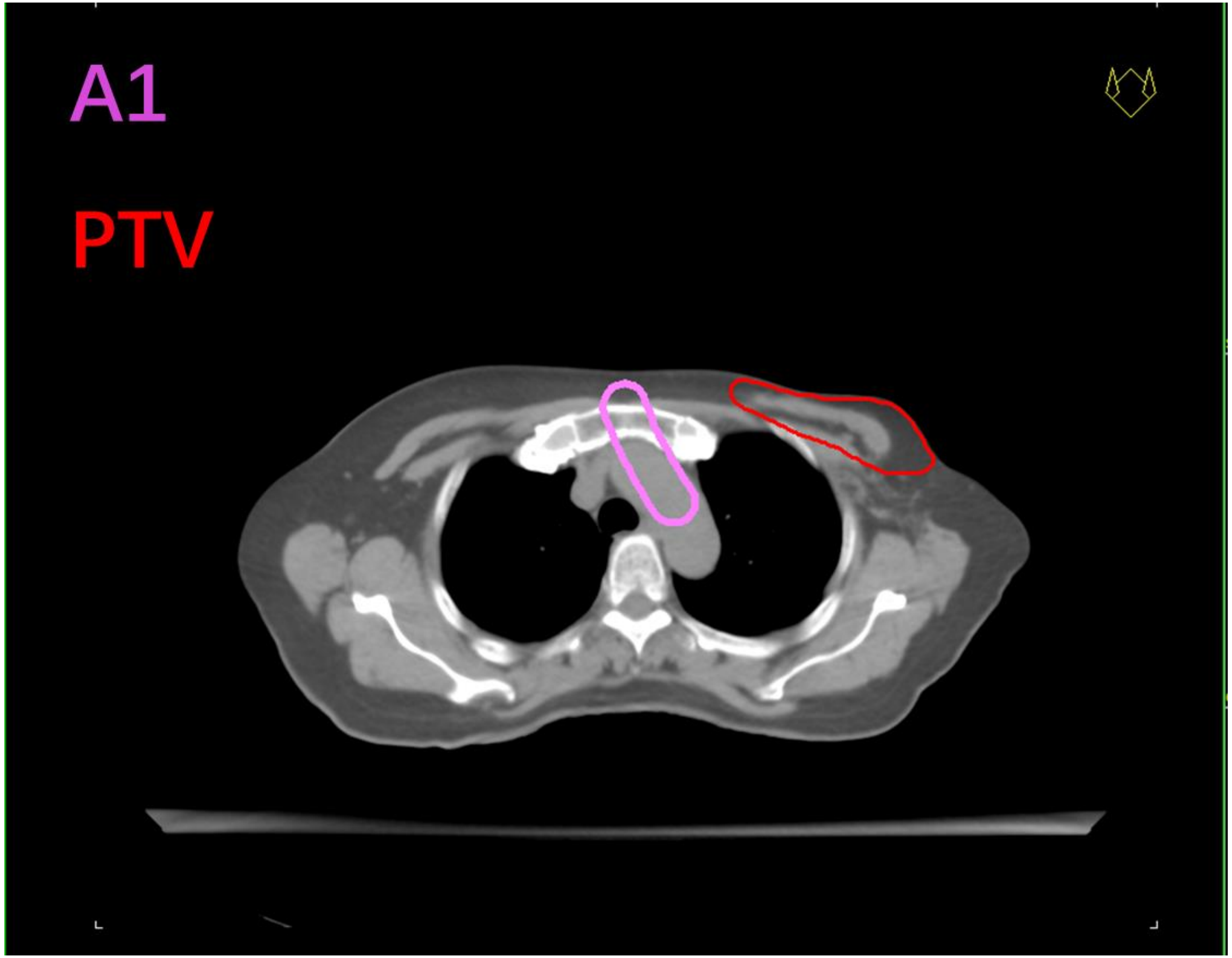
A1
PTV

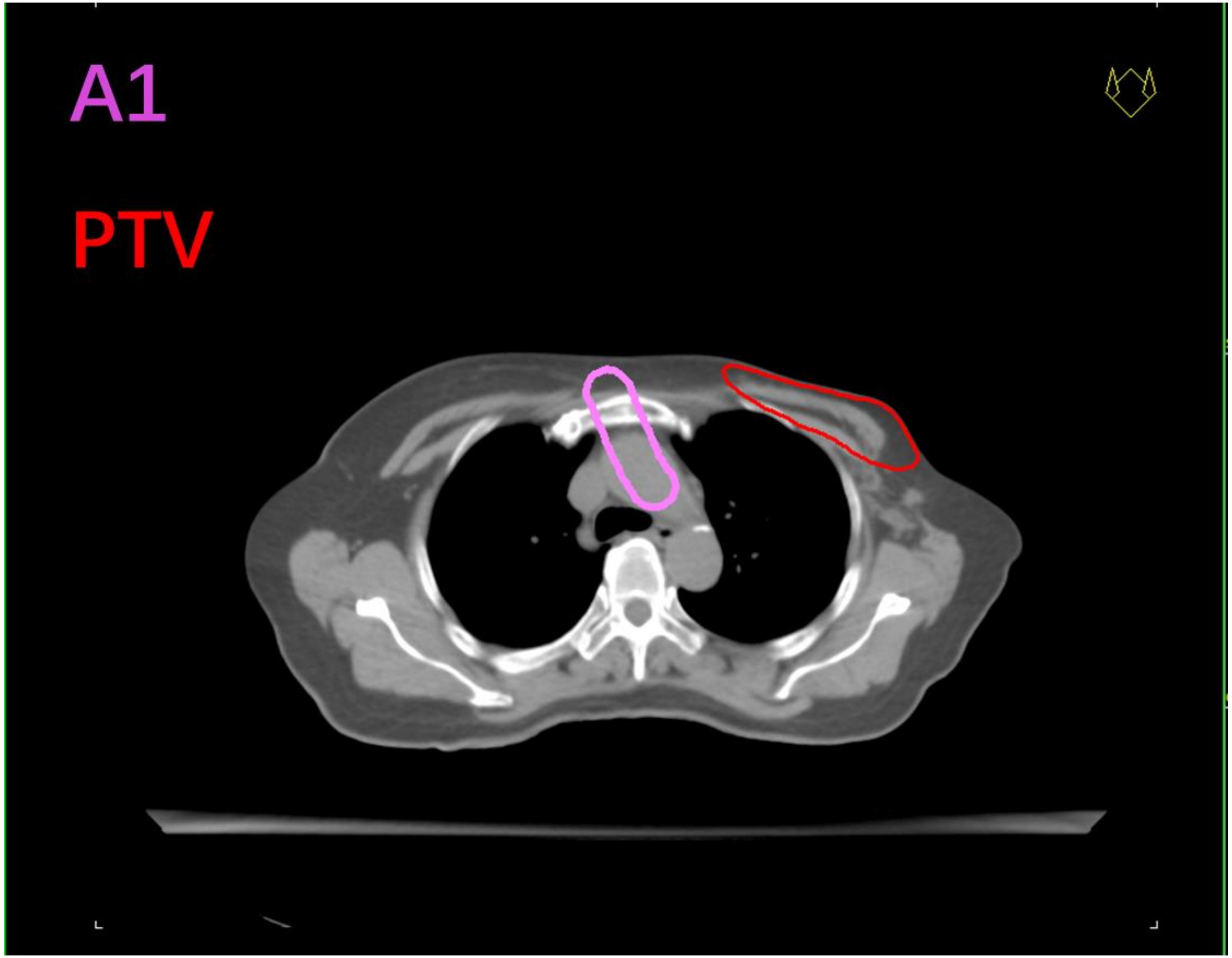
A1
PTV

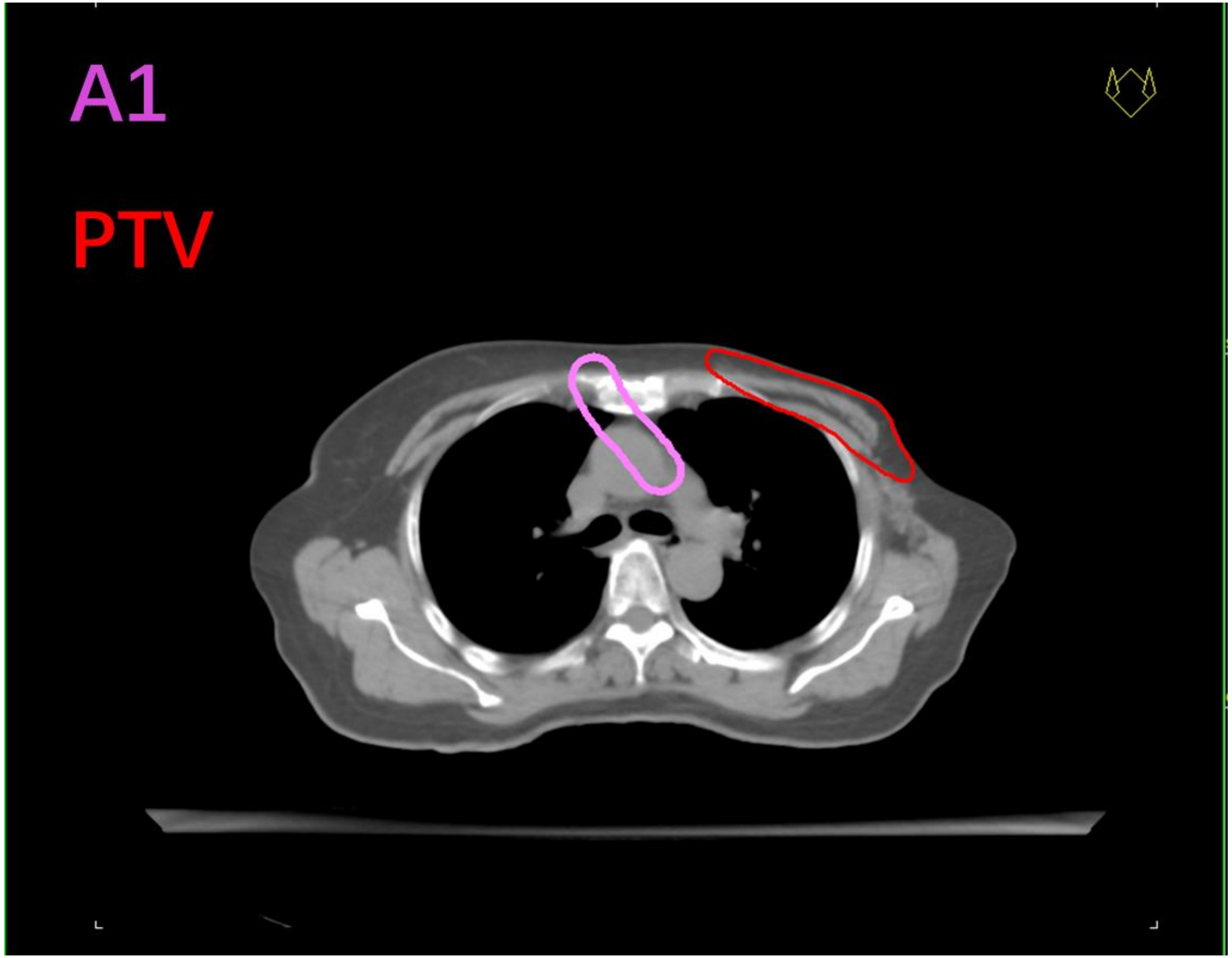
A1
PTV

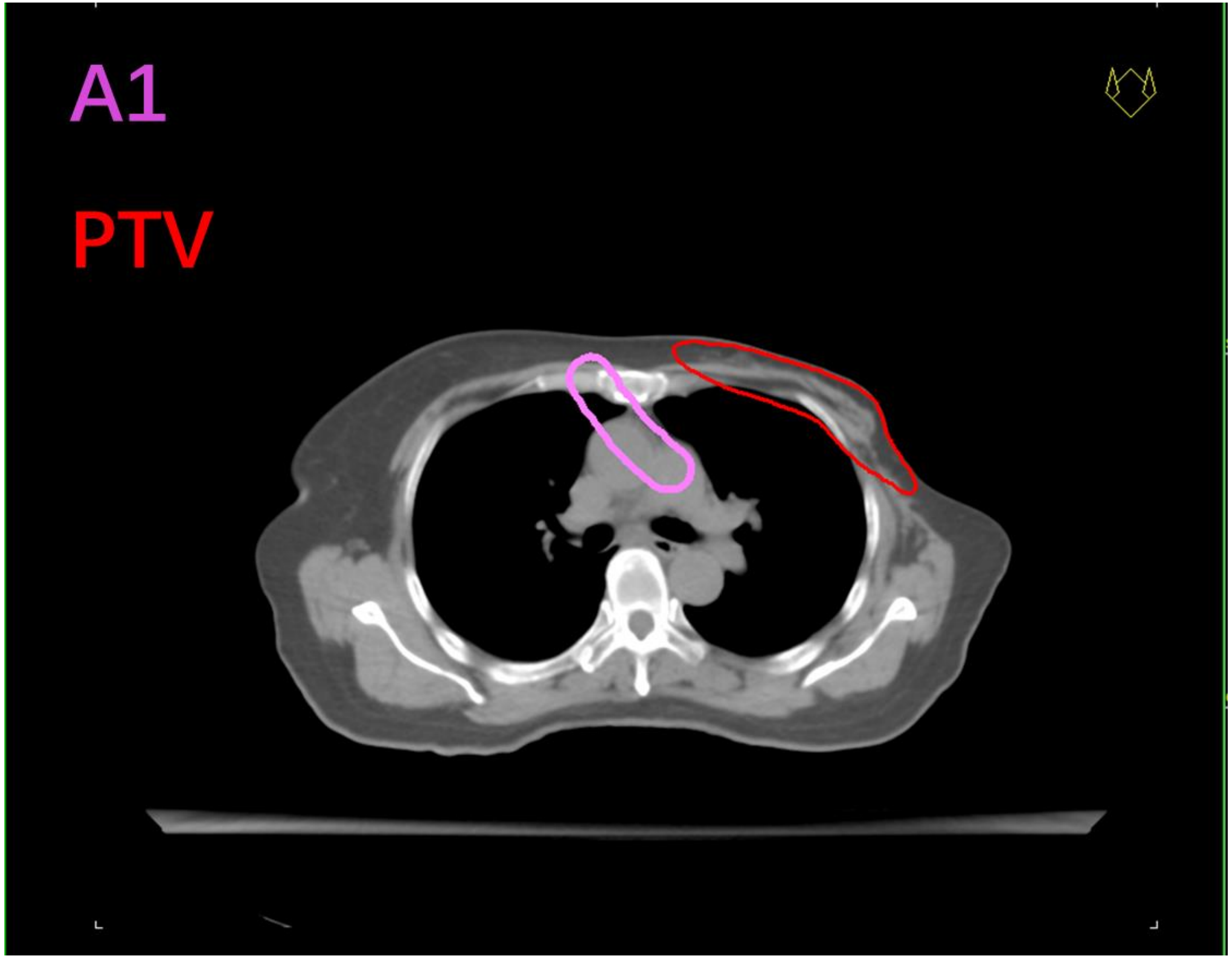
A1
PTV

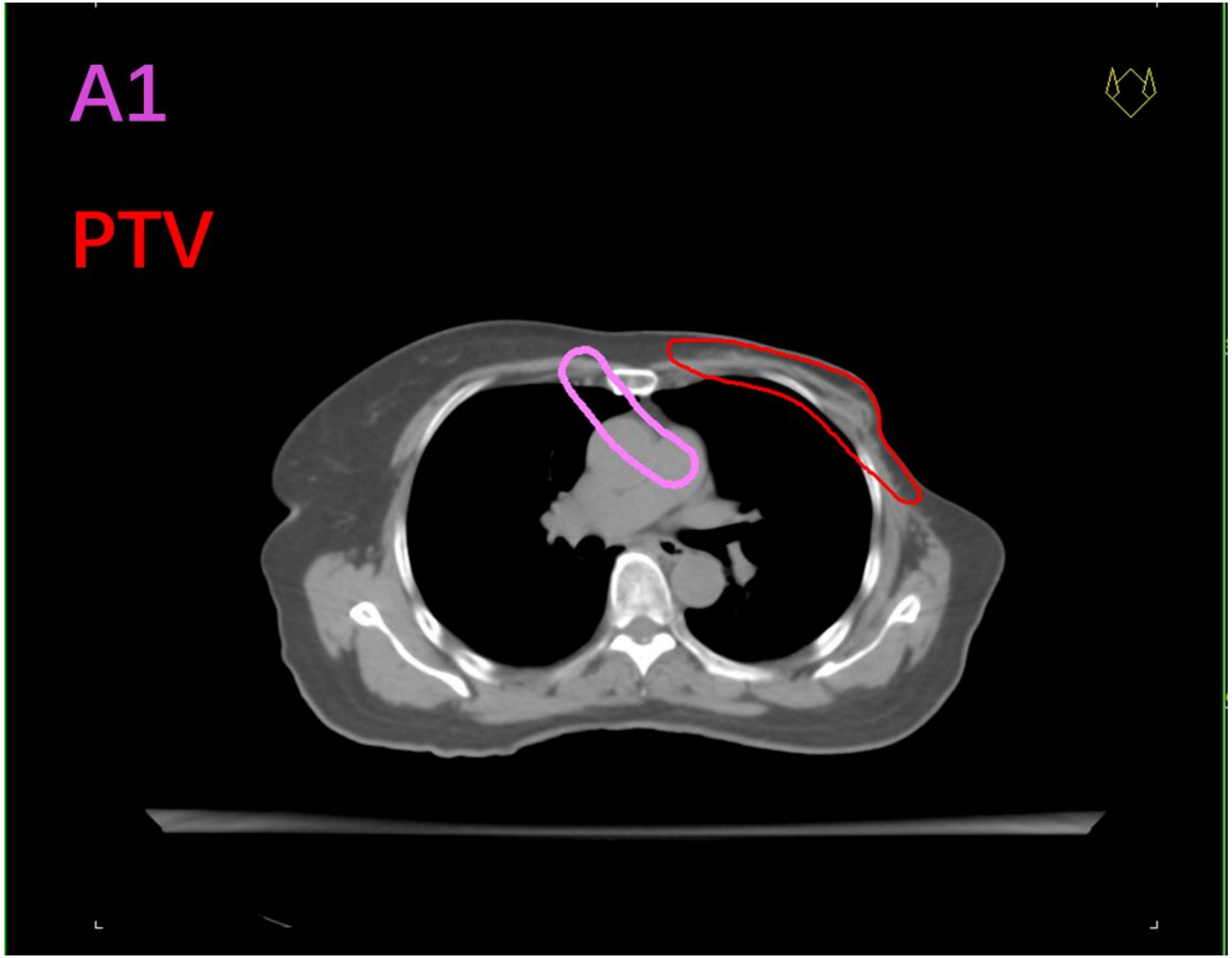
A1
PTV

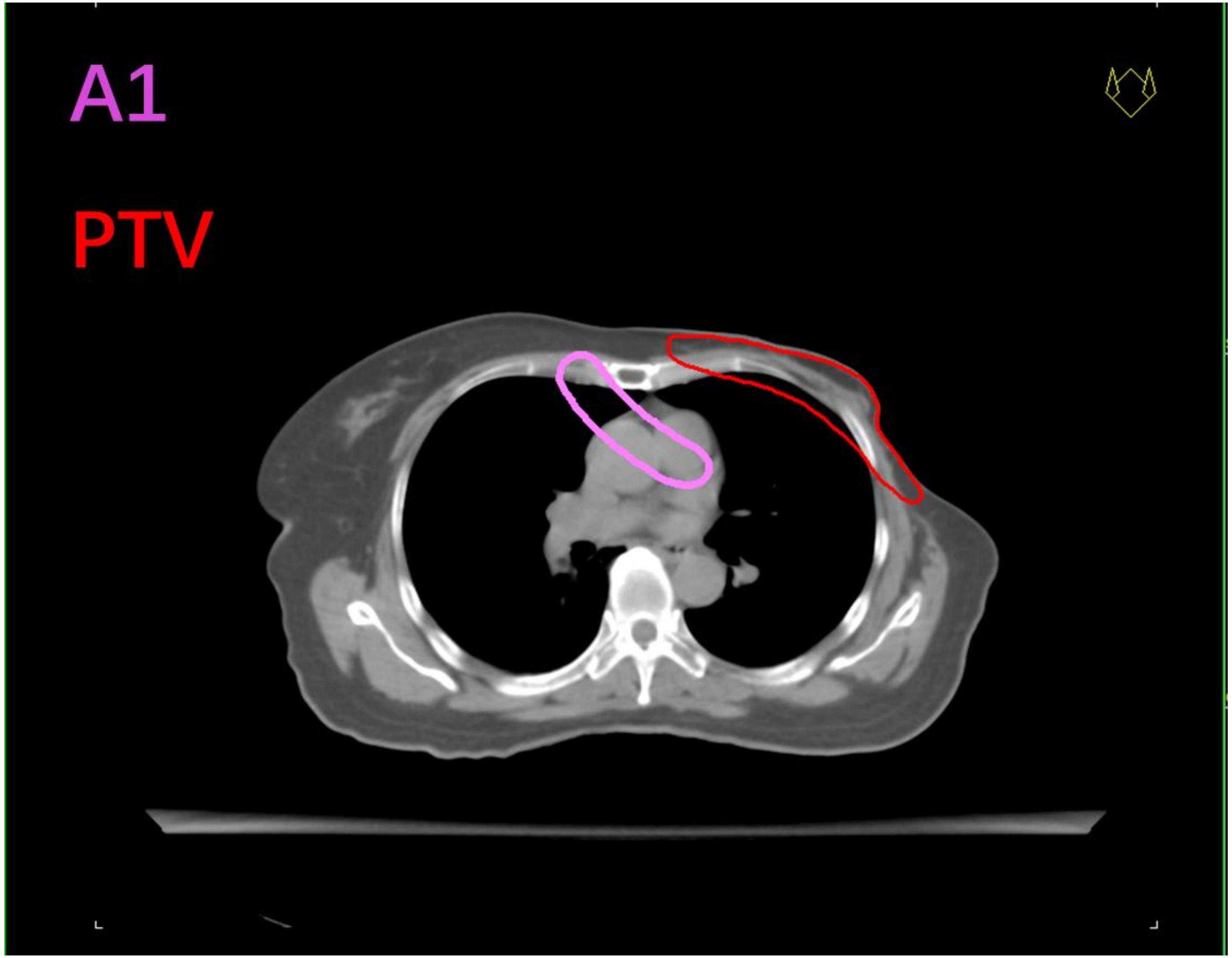
A1
PTV

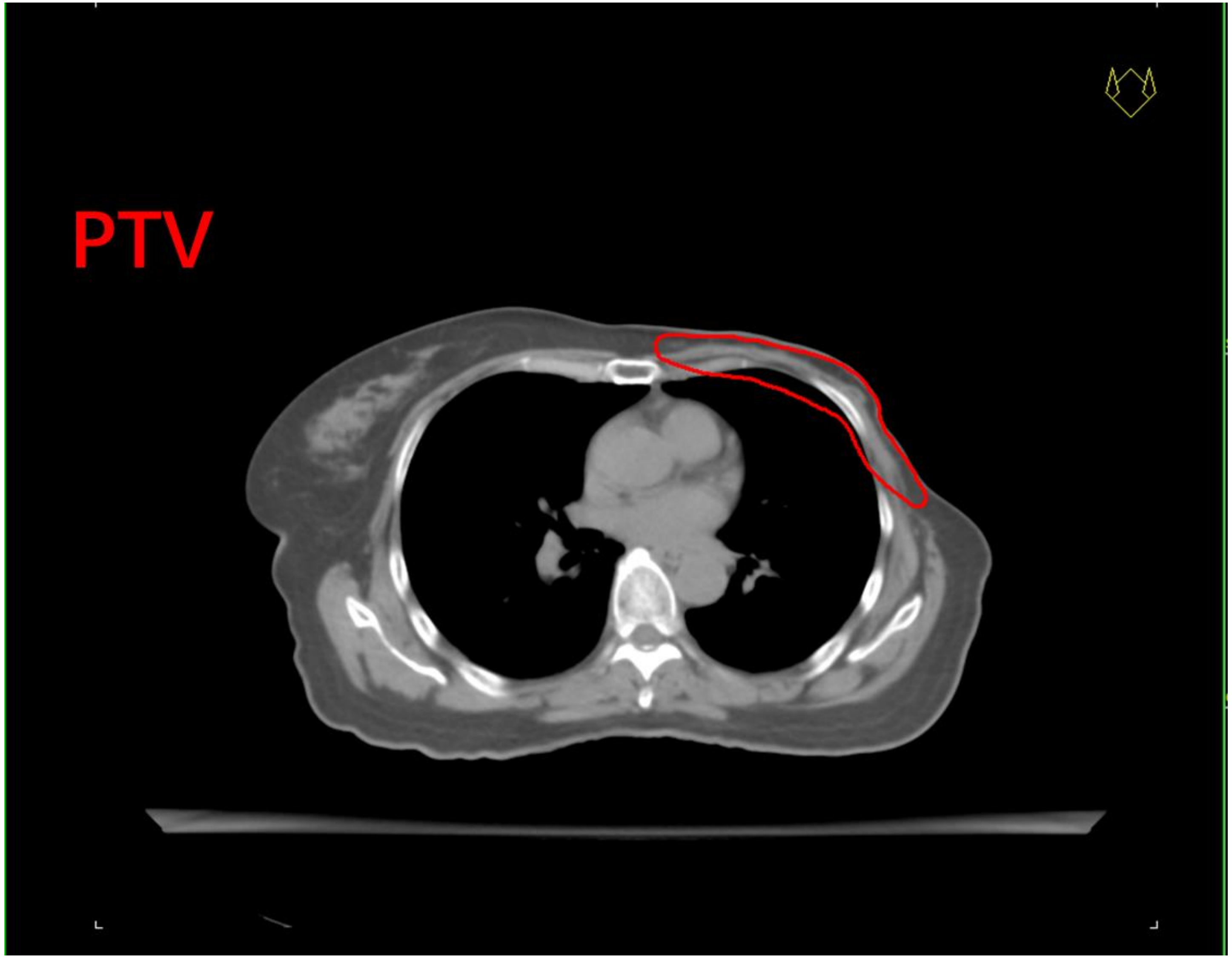
PTV

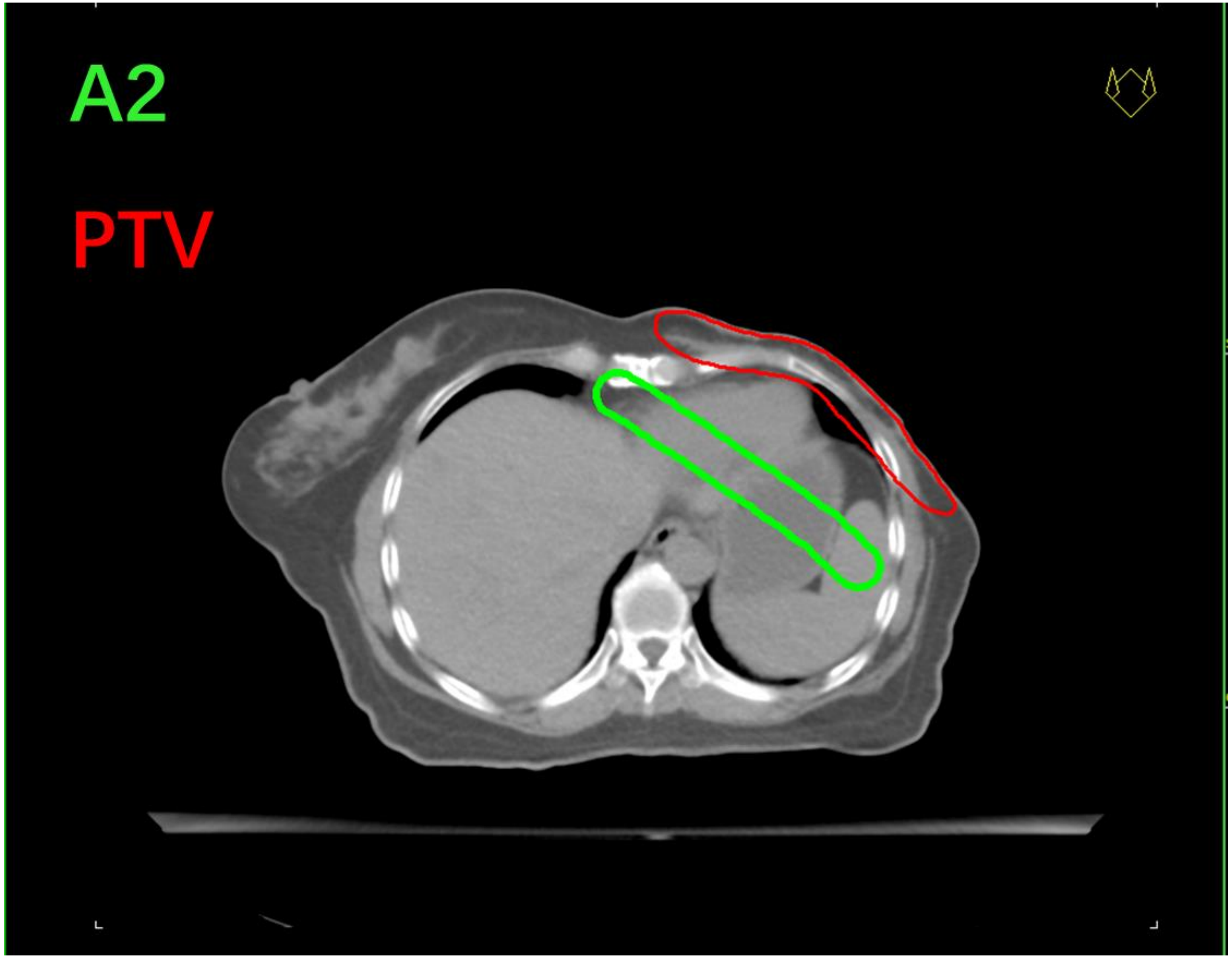
A2
PTV

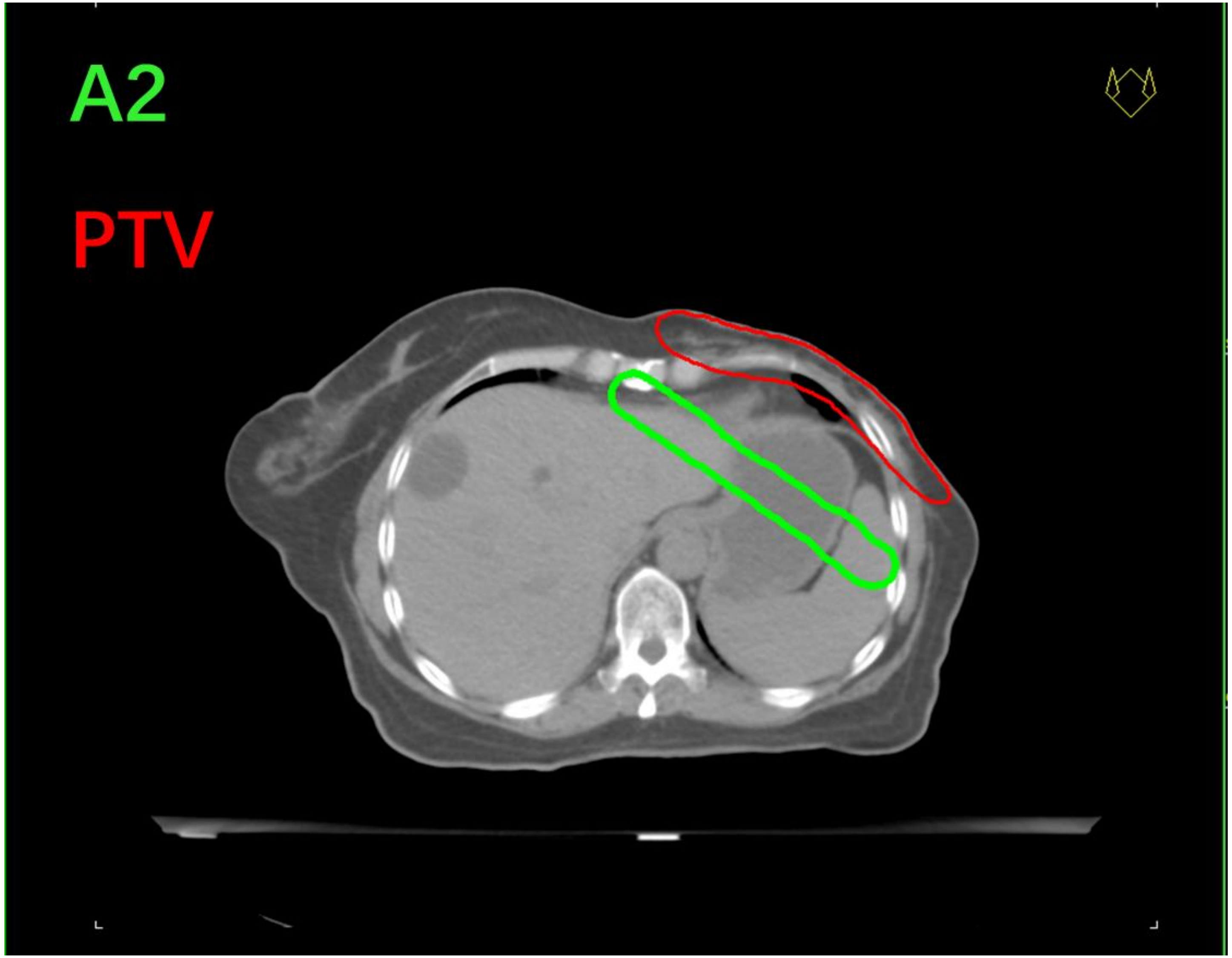
A2
PTV

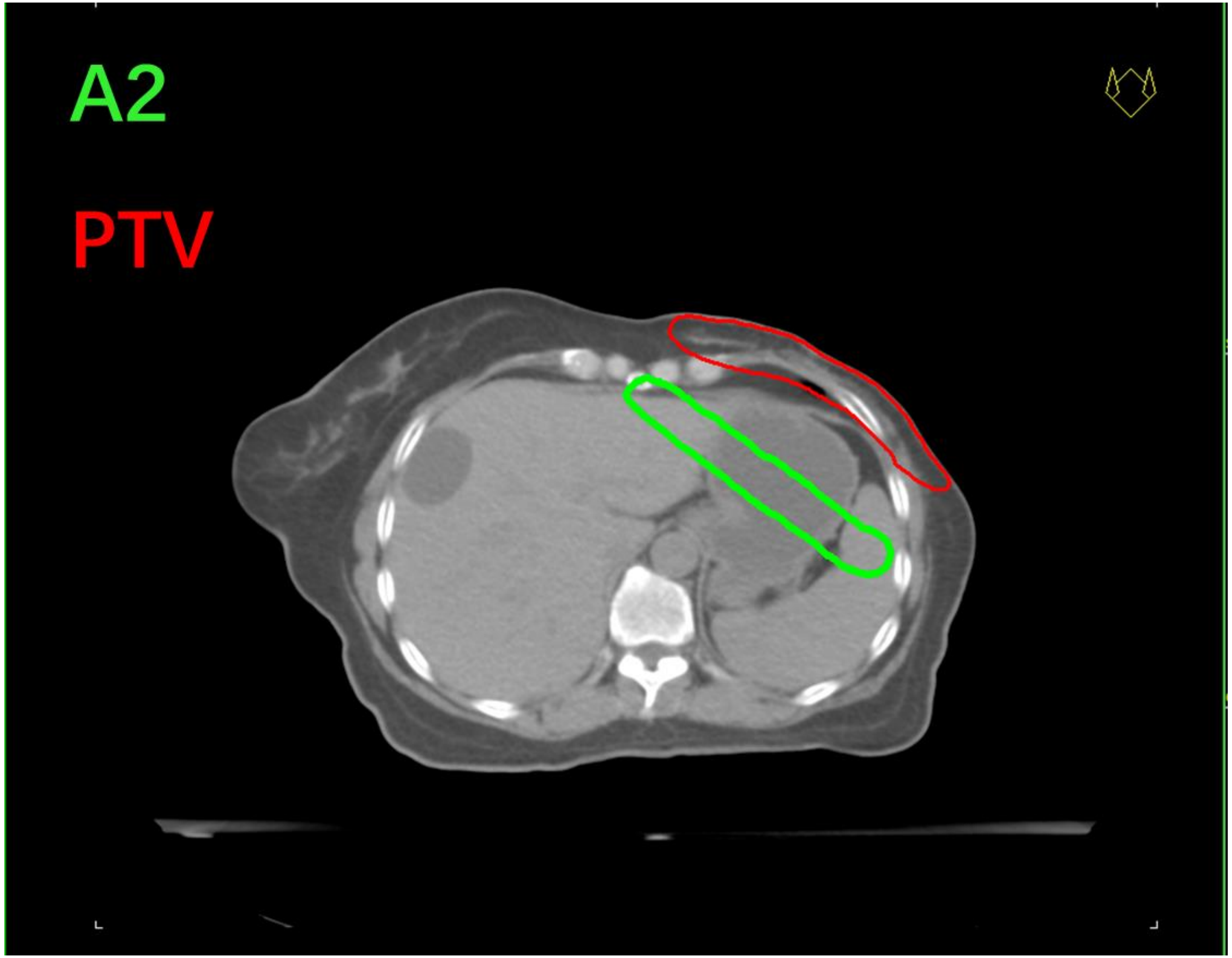
A2
PTV

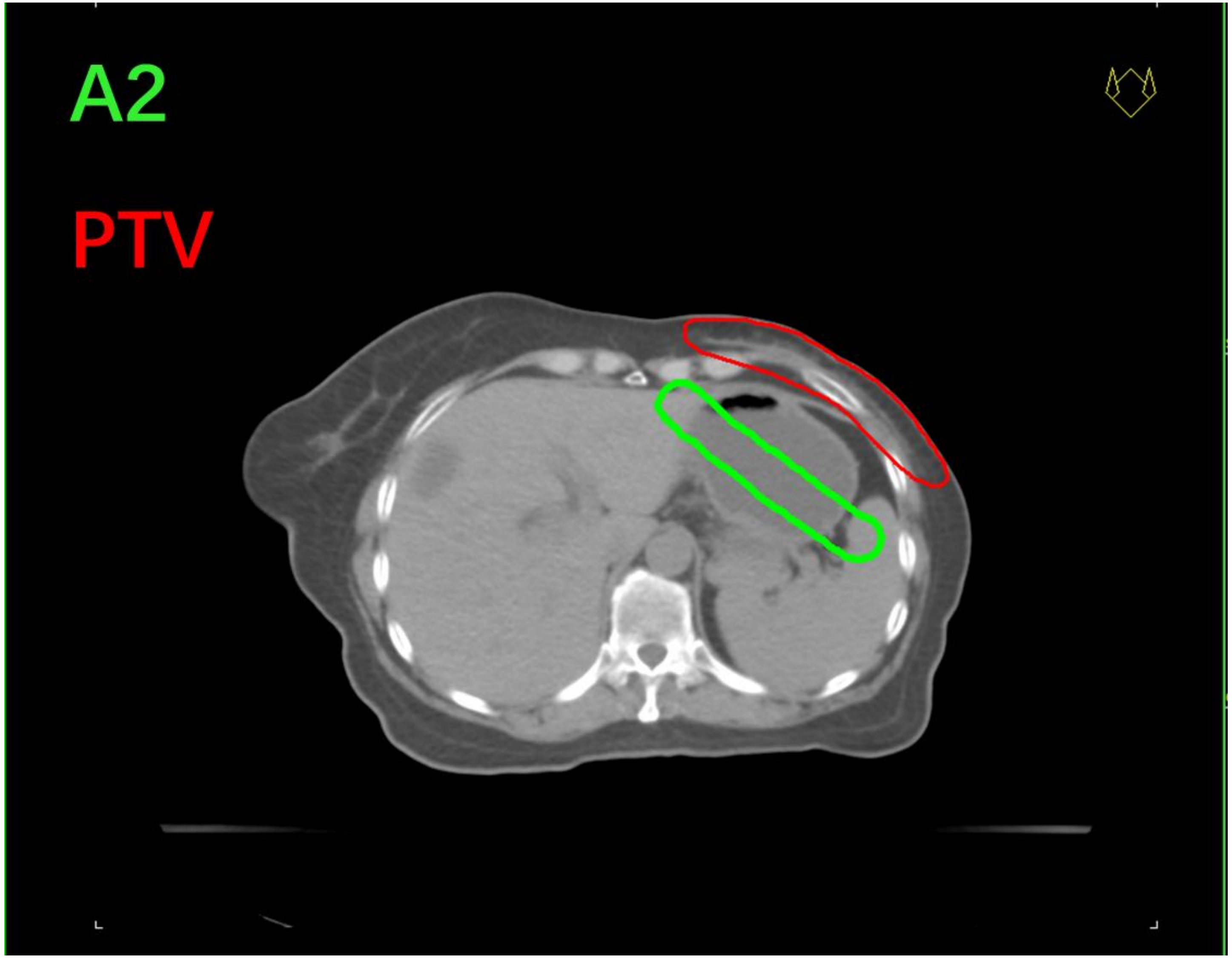
A2
PTV

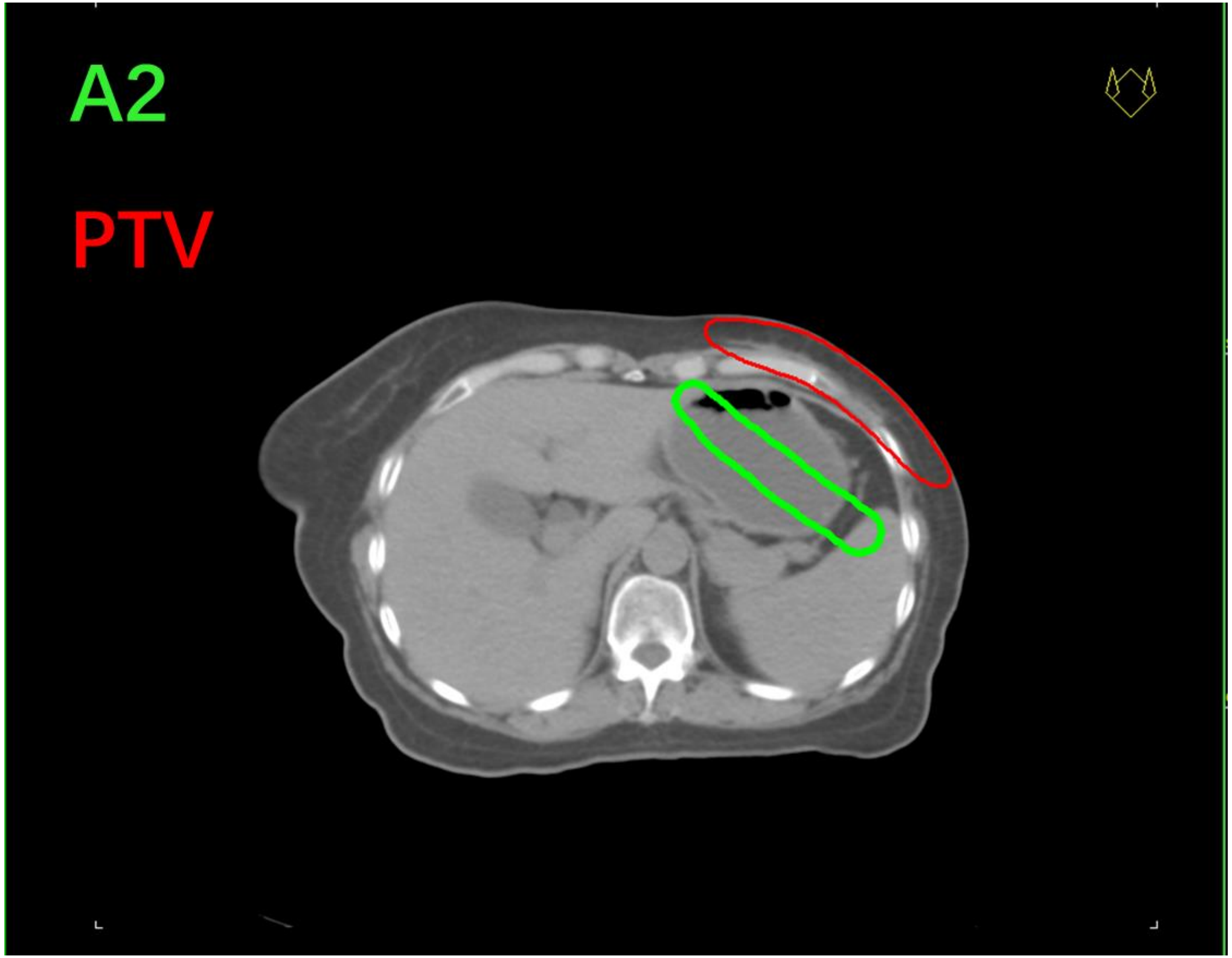
A2
PTV

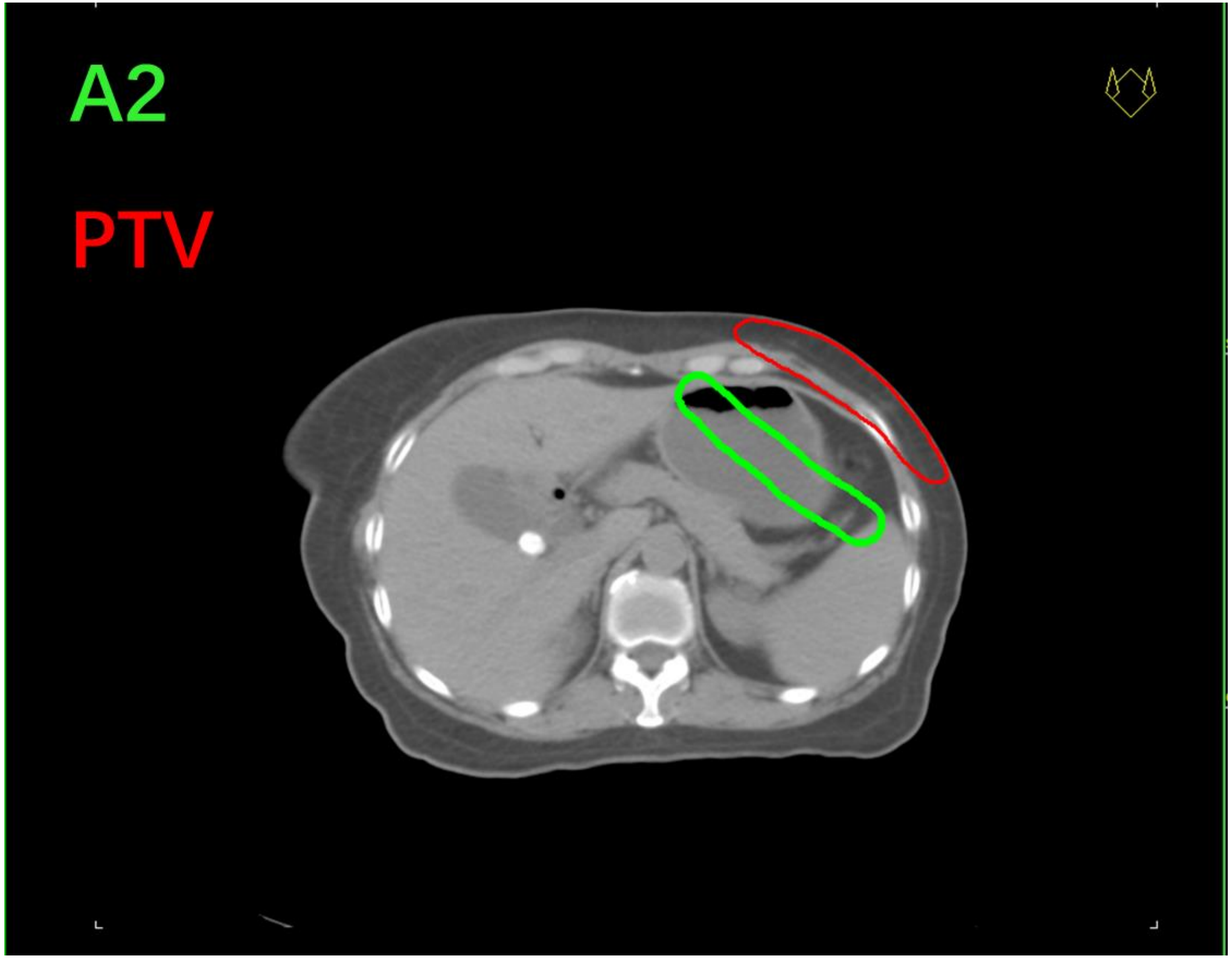
A2
PTV

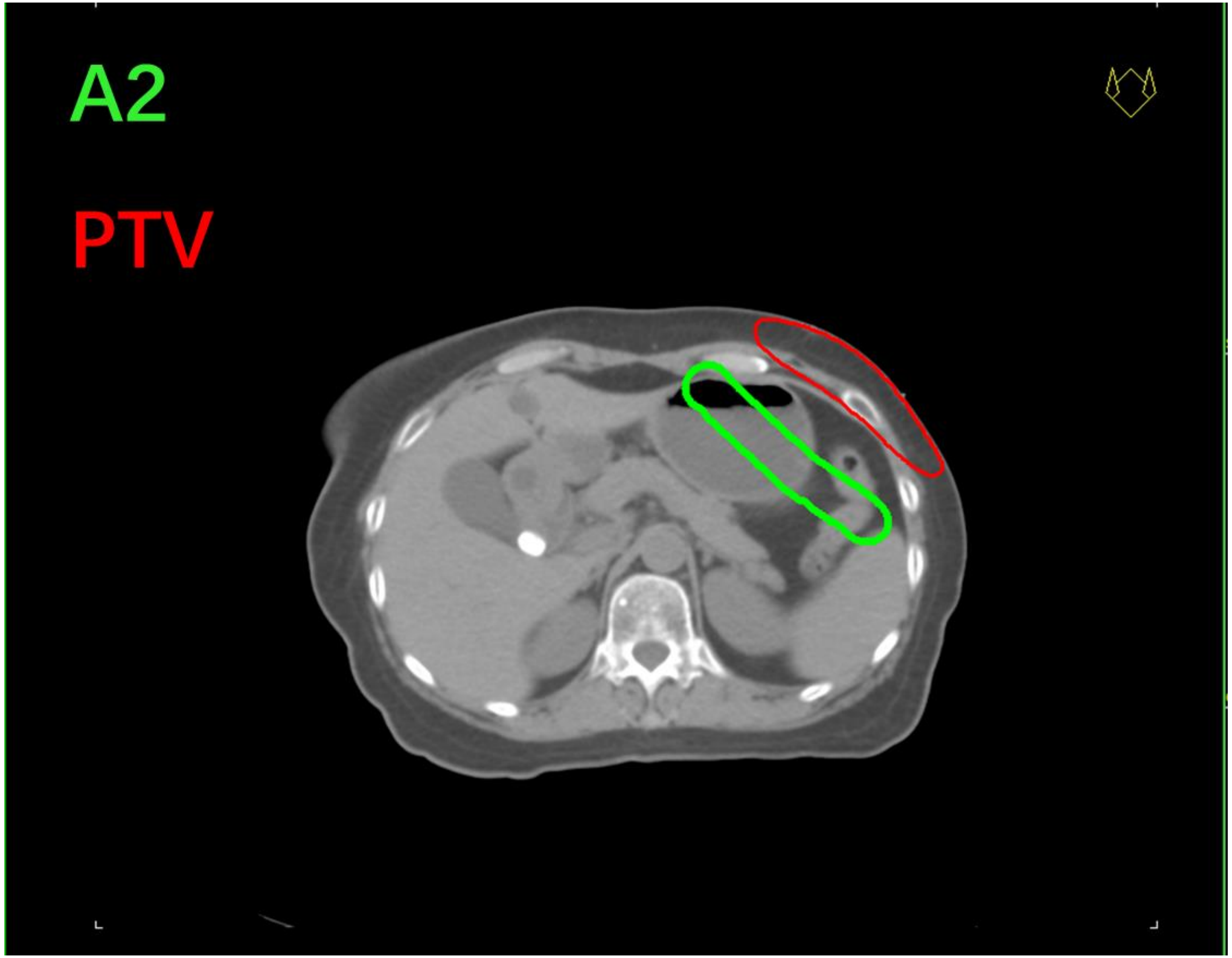
A2
PTV

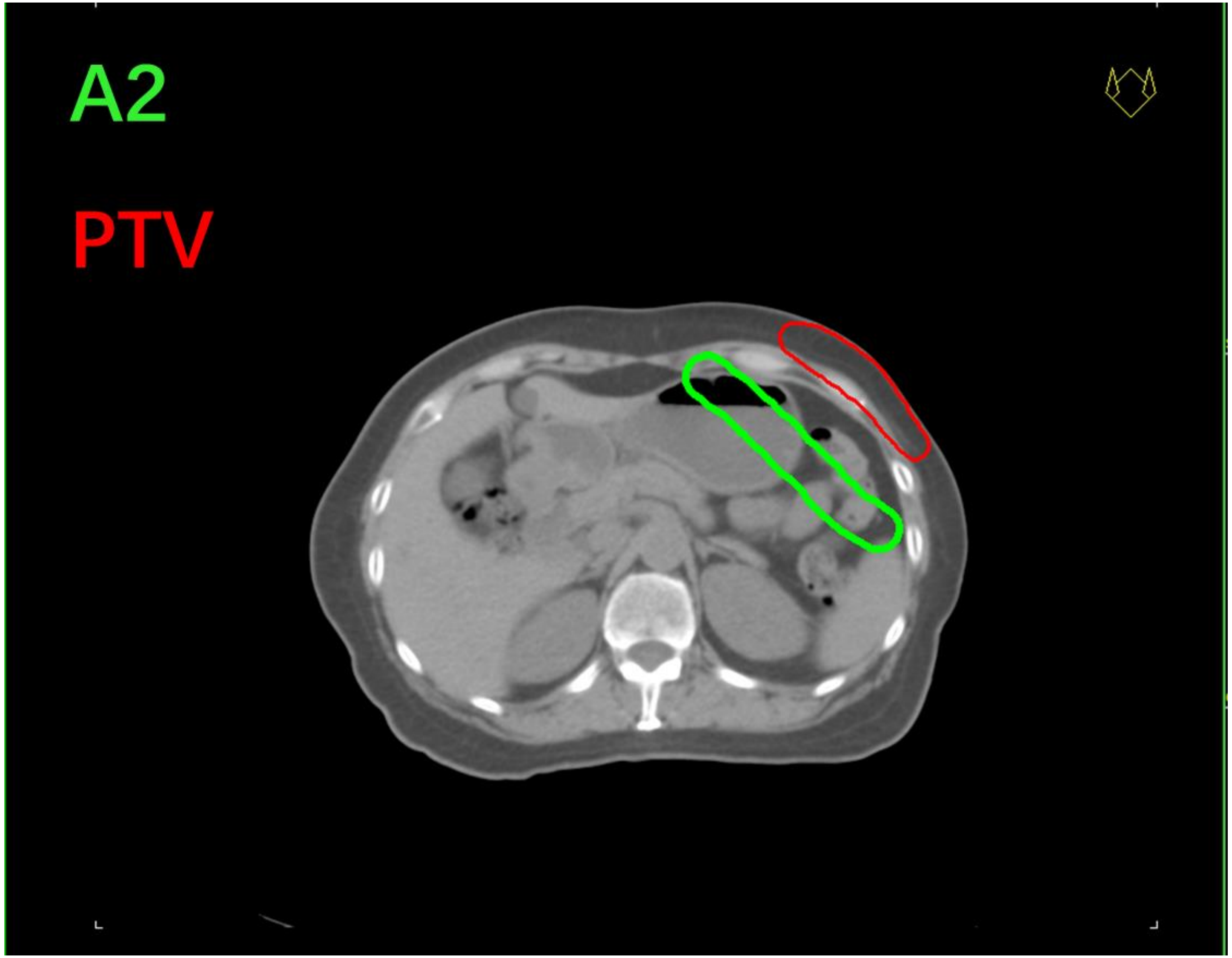
A2
PTV

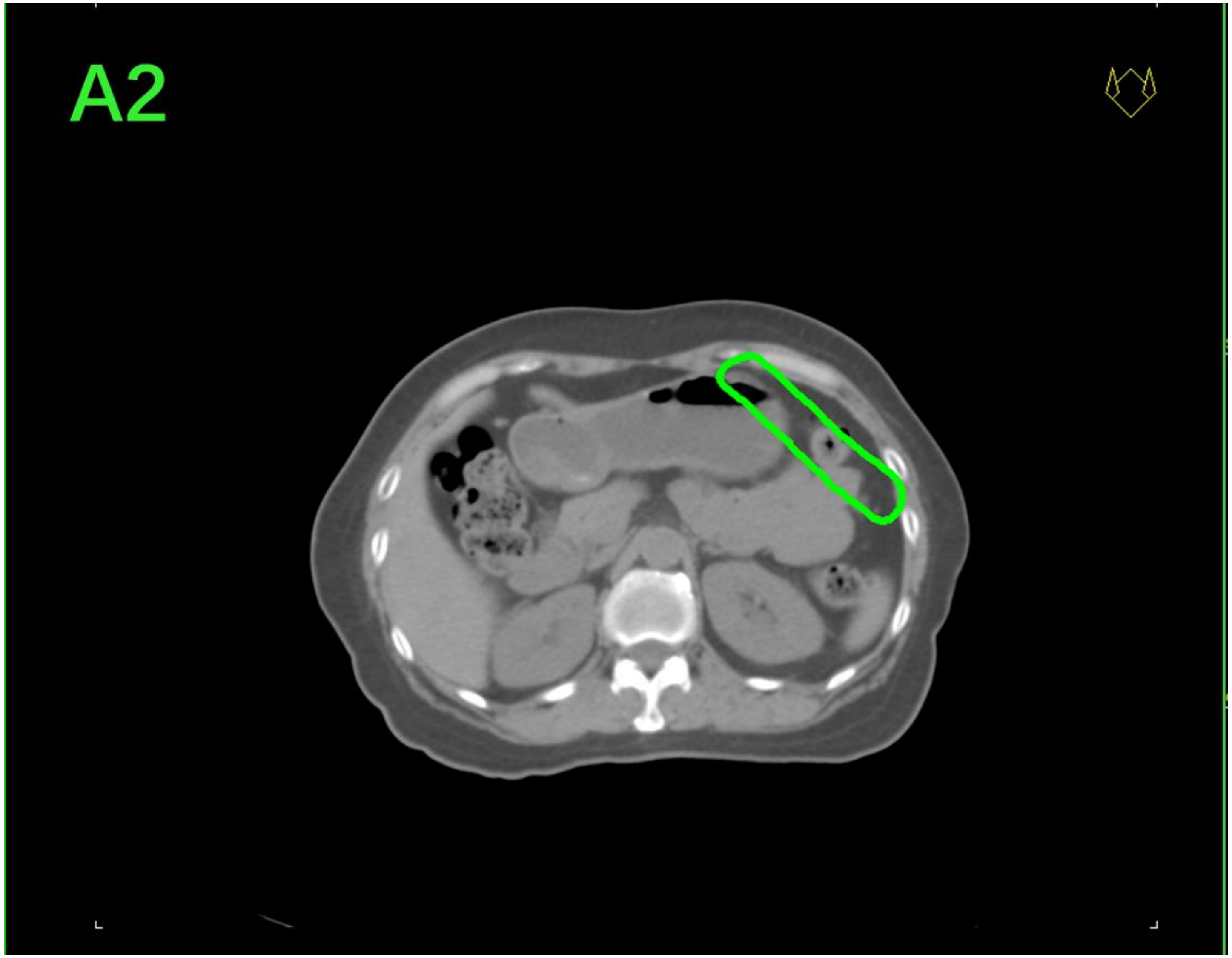
A2
